# Selection and validation of reference genes for normalisation of gene expression in ischaemic and toxicological studies in kidney disease

**DOI:** 10.1371/journal.pone.0233109

**Published:** 2020-05-21

**Authors:** Sanjeeva Herath, Hongying Dai, Jonathan Erlich, Amy YM Au, Kylie Taylor, Lena Succar, Zoltán H. Endre

**Affiliations:** 1 Prince of Wales Clinical School, University of New South Wales, Randwick, New South Wales, Australia; 2 Department of Biostatistics, College of Public Health, University of Nebraska Medical Center, Omaha, Nebraska, United States of America; 3 Department of Nephrology, Prince of Wales Hospital, Randwick, New South Wales, Australia; University Medical Center Utrecht, NETHERLANDS

## Abstract

Normalisation to standard reference gene(s) is essential for quantitative real-time polymerase chain reaction (RT-qPCR) to obtain reproducible and comparable results of a gene of interest (GOI) between subjects and under varying experimental conditions. There is limited evidence to support selection of the commonly used reference genes in rat ischaemic and toxicological kidney models. Employing these models, we determined the most stable reference genes by comparing 4 standard methods (NormFinder, qBase+, BestKeeper and comparative ΔCq) and developed a new 3-way linear mixed-effects model for evaluation of reference gene stability. This new technique utilises the intra-class correlation coefficient as the stability measure for multiple continuous and categorical covariates when determining the optimum normalisation factor. The model also determines confidence intervals for each candidate normalisation gene to facilitate selection and allow sample size calculation for designing experiments to identify reference genes. Of the 10 candidate reference genes tested, the geometric mean of polyadenylate-binding nuclear protein 1 (*PABPN1*) and beta-actin (*ACTB*) was the most stable reference combination. In contrast, commonly used ribosomal *18S* and glyceraldehyde 3-phosphate dehydrogenase (*GAPDH*) were the most unstable. We compared the use of *PABPN1×ACTB* and 2 commonly used genes *18S* and *GAPDH* on the expression of 4 genes of interest know to vary after renal injury and expressed by different kidney cell types (*KIM-1*, *HIF1α*, *TGFβ1* and *PECAM1*). The less stable reference genes gave varying patterns of GOI expression in contrast to the use of the least unstable reference *PABPN1×ACTB* combination; this improved detection of differences in gene expression between experimental groups. Reduced within-group variation of the now more accurately normalised GOI may allow for reduced experimental group size particularly for comparison between various models. This objective selection of stable reference genes increased the reliability of comparisons within and between experimental groups.

## Introduction

Quantitative real-time polymerase chain reaction (RT-qPCR) is a widely used, sensitive and specific method for detection and quantification of messenger RNA (mRNA) expression over a large dynamic range for validation of selected genes identified by other techniques (e.g. RNA-seq) [1] [2]. For rare mRNA species, RT-qPCR may be the only practical way to quantitate gene expression. A critical aspect of RT-qPCR assessment of gene expression is controlling for the amount of starting material, i.e., normalisation of gene expression to an endogenous gene not affected by the experimental conditions. Normalisation is critical for comparison of samples from different sources that may contain varying quantities of mRNA [3]. Differences in the quantity of mRNA may result from differences in extraction and isolation efficiency of mRNA species. Variation in input quantity to RT reactions and inefficiency in complimentary deoxyribose nucleic acid (cDNA) synthesis may also introduce experimental variation [4]. For this study, these sources of variation were termed ‘experimental error’. Varying normalisation strategies have been used to minimise the intrinsic variability resulting from such sources. Techniques include normalisation to the initial total RNA, addition of known quantities of cDNA, and the use of internal reference genes [4, 5]. Endogenous reference genes are regarded as optimal, since detection and amplification of reference genes and target genes occur under the same conditions [6]. Nevertheless, critical prerequisites for an ideal reference gene are a broad dynamic range and constant level of expression compared to the gene of interest (GOI) under the same experimental conditions and, ideally, under different experimental conditions [7, 8]. Selection of appropriate reference genes is critical to reducing experimental error. However, there is no consensus regarding the best reference genes based on rat strain, tissue type and injury model with a variety of genes suggested for various organs or regions of various organs [9–16].

Housekeeping genes (HKGs) are ideal candidate reference genes as these are constitutive genes required for basic cellular function and expressed in most cells under normal physiological conditions [9]. However, HKGs may be affected by experimental conditions as many represent metabolic pathways or structural genes that may be altered by experimental interventions [10]. Commonly employed HKGs for RT-qPCR include beta actin (*ACTB*), glyceraldehyde 3-phosphate dehydrogenase (*GAPDH*) and 18S ribosomal RNA (*18S*). The choice of these mRNA species stems from use in traditional non- or semi-quantitative methods such as Northern blotting and have often not been validated for RT-qPCR across diverse experimental conditions [8]. A growing body of evidence suggests that, both *in vivo* and *in vitro*, there may be considerable fluctuation under varying experimental conditions making them unsuitable as reference genes for RT-qPCR [2, 10, 12, 17–21]. For example, *GAPDH* varies under hypoxic stress [13] and *18S* varies under both toxic and hypoxic stress [2]. In addition, reference genes may be tissue specific, so that ideal HKGs may differ between tissues and experimental conditions [22, 23]. Currently, normalisation against the geometric mean of the most stable reference genes is regarded as the best strategy for error reduction in raw qPCR data [24].

A number of statistical algorithms such as Normfinder [11], geNorm (14, improved to qBase+), BestKeeper [25, 26] and comparative delta quantification cycle (ΔCq) approach [27] were devised to evaluate stable reference gene/s for given experimental conditions. While these represent an improvement over arbitrary selection, there are limitations to these algorithms. They do not allow for randomly missing data and do not account for multiple systemic effects (experimental conditions), systemic effects related to continuous variables, or for reference gene-systemic effect interactions. In addition, stability values are not accompanied by confidence intervals (CIs). This precludes determination of minimal sample size, which could reduce error in reference gene selection. It also prevents ranking of reference genes with close stability values. In order to address these limitations a 3-way linear mixed-effects model (LMM) was employed based on the intra class correlation coefficient (ICC) [28].

The rat is one of the most commonly used models in the study of renal disease [29]. Ischaemia-reperfusion injury, IRI, and toxic injury represent the majority of causes of acute kidney injury [30]. However, pre-clinical animal studies have been plagued by inconsistent, poorly reproducible results. Gene expression studies often guide pre-clinical studies and direct further investigation. A consistent method of normalising GOI expression between experimental groups in time and across treatment strategies is critical. Thus, validated stable endogenous reference genes are needed to normalise RT-qPCR data for rat kidney and other studies.

There has been an increase in the number of studies evaluating the stability of gene expression in various tissues, however, few have studied the reference genes in rodent kidney. Validation studies of reference genes in rodents have been performed mainly in liver and heart injury models, usually for ischaemic and toxic injury [2, 12, 13, 31]. We selected 10 candidate genes for analysis based on an exhaustive literature review of rat studies performed over the last 20 years to evaluate the suitability of reference genes. Table 1 summarises the most pertinent of these rat studies and compares these with human studies.

**Table 1:** Reference gene evaluation studies.

Number of reference genes studied	Pathology/Intervention/treatment	Tissue	Species	Reference Gene Suitability	Weight when selecting Reference genes	Reference
3	Various acute and chronic kidney pathologies	Kidney	Human	*18S* and *GAPDH* unstable and unsuitable to be used singly as reference genes	4	[32]
10	Hypoxia	Kidney HEK cell line	Human	The most suitable reference genes for RT-qPCR studies in kidney HEK cell line were *Ppia*, *HPRT*, *B2M*	4	[33]
16	Cystic kidney disease	Kidney	Mouse	The most suitable reference genes were *Ppia*, *GAPDH*, *Pgk1*	4	[31]
6	Influence of testosterone on kidney (orchidectomy +/- testosterone)	Kidney	Rat (Sprague-Dawley)	Most suitable and stable normalisation factor for RT-qPCR studies in kidney was *HMBS + GAPDH*	5	[10]
10	Fasting and acute hyperglycaemia	Kidney	Rat (Zucker)	The most suitable reference genes RT-qPCR studies in kidney was *TBP*, *ACTB*, *GAPDH*	5	[9]
10	Post infarction heart failure	Myocardium	Human	The most suitable reference genes in humans were *Rpl32* and *Pgk1*	3	[13]
10	Post infarction heart failure	Myocardium	Mouse	The most stable reference genes in mice *Rpl32*, *GAPDH* and *Polr2a*	3	[13]
10	Post infarction heart failure	Myocardium	Rat (Wistar)	The most suitable reference genes in *Polr2a*, *Rpl32* and *TBP*	4	[13]
9	Ischaemia reperfusion	Heart	Rat (Wistar)	For RV were *HMBS* + *HPRT*	4	[12]
				For LV were *YWHAZ+ PABPN1+HMBS*	4	
10	Fasting and acute hyperglycaemia	Heart	Rat (Zucker)	Most stable was *SDHA*, *TBP*	4	[9]
2	Asthma	Endobronchial tissue and bronchoalveolar lavage cells	Human	*GAPDH*, *ACTB* unsuitable as reference genes	2	[34]
10	Fasting and acute hyperglycaemia	Lung	Rat (Zucker)	Most stable were *ACTB*, *YWHAG*	3	[9]
12	Control (no insult)	Juvenile and adult rat tissue (ovary, liver, adrenal, prostate, fat pad, testis)	Rat (Wistar)	Most stable for both adult and juvenile rat tissues (i.e across developmental stages) were *HPRT* and *SDHA*	3	[2]
12	Various toxicological insults	Liver, ovary, adrenal, prostate, fat pad, testis	Rat (Wistar)	Most stable reference gene in all tissues examined were *HPRT* and *SDHA*	3	[2]
8	Hepatotoxicity	Liver	Rat (Wistar)	The most suitable reference genes *SDHA* and *r18S*	3	[6]
17	Fat gavage or dietary restriction	Liver	Rat (Wistar)	The most suitable reference genes for RT-qPCR studies in liver was r*18S*	3	[35]
17	Fat gavage or dietary restriction	Duodenum, jejunum, ileum	Rat (Wistar)	The most suitable reference genes for RT-qPCR studies in duodenum was *TBP*	3	[35]
				In jejunum was *Ubc*	3	
				In ileum was *HPRT*	3	
6	Influence of testosterone on hypothalamus (orchidectomy +/- testosterone)	Hypothalamus	Rat (Sprague-Dawley)	The most table reference genes were *HMBS* and *Ppia*	3	[10]
10	Hypoxia	Breast MCF 7 cell line	Human	Most stable reference genes in MCF 7 cell line were *TBP* and *ATP5G3*	1	[33]
10	Hypoxia	Prostate LNCaP and PNT2	Human	For LNCaP cell lines *GAPDH* and *TBP* and PNT2 cell line were *ATP5G3* and *HPRT*	1	[33]
10	Cell Culture	Cultured T helper cells	Human	*GAPDH* unstable and *MLN51*, *EF-1-α*, *UbcH5* more stable and suitable	1	[36]
13	Cell Culture, TB	Culture of whole blood and PBMC	Human	*HuPO* most stable and suitable in whole blood	1	[18]
				*HuPO* and *HPRT* most stable suitable in cultured PBMC	1	
9	Differentiation of intestinal and colonic adenocarcinoma	Cultured intestinal epithelial cells	Human	The most suitable reference genes for RT-qPCR studies in differentiating intestinal epithelial was *RPLPO*	1	[37]
				In adenocarcinoma of colon was *B2M*	1	
6	Normoxia, chronic hypoxia or hyperoxia	Early post-natal period carotid body	Rat (Sprague-Dawley)	*Ppia* + *TBP* most stable and suitable normalisation factor overall	3	[38]

*ACTB* = beta-actin*; ATP5G3* = ATP synthase, H+ transporting, mitochondrial F0 complex, subunit C3 (subunit 9); *B2M* = beta-2-microglobulin*; EF-1-a* = elongation factor 1-alpha; *GAPDH* = glyceraldehyde-3-phosphate dehydrogenase; *HMBS* = hydroxymethylbilane synthase; *HPRT* = hypoxanthine phosphoribosyltransferase; *HuPO* = human acidic ribosomal protein; *PABPN1* poly(A) binding protein nuclear 1; PBMC = peripheral blood mononuclear cells; *Pgk1* = phosphoglycerate kinase 1; *Polr2a* = RNA polymerase II subunit A; Ppia = peptidylprolyl isomerase A; *r18S* = ribosomal 18S subunit; *Rpl32* = ribosomal protein L32; *RPLPO* = ribosomal phosphoprotein; *SDHA* = succinate dehydrogenase complex flavoprotein subunit A; TB = tuberculosis; *TBP* = TATA-box binding protein; *UbcH5B* = ubiquitin-conjugating enzyme E2 D2; *YWHAG* = tyrosine 3-monooxygenase/tryptophan 5-monooxygenase activation protein gamma; *YWHAZ* = tyrosine 3-monooxygenase/tryptophan 5-monooxygenase activation protein zeta

Experimental conditions which overlapped with the present study were weighted to a greater extent. Hence studies concerned with rat and kidney models were weighted to a greater extent than those of IRI which in turn were weighted to a greater extent than the rest of the studies. An arbitrary scale out of 5 was used to weigh each study when selecting 10 candidate reference genes for the present study based on this criteria and is detailed in Table 1. *Ppia* and *Polr2a* were excluded from analysis as although they have been identified as stable in myocardial and carotid body IRI models [13, 38], the aforementioned *Ppia* and *Polr2a* genes were shown to be the most unstable in kidney tissue in a study analysing reference genes in obese zucker rats [9].

The primary aim was to determine the most stable reference genes for normalisation of gene expression studies in ischaemic and toxic rat renal kidney injury models. A second aim was to compare the results of the current standard techniques with that of a 3-way LMM and to estimate the sample size for determination of accurate qPCR results for given combinations of reference genes with known ICCs. Kidney injury molecule (*KIM*)*-1*, Hypoxia Inducible Factor 1 Subunit Alpha (*HIF1α*), Platelet and Endothelial Cell Adhesion Molecule 1 (*PECAM1*), Transforming Growth Factor Beta 1 (*TGFβ1*), were assayed as representative GOIs to allow statistical comparisons.

## Results

### Descriptive statistics and gene expression

**Fig 1:**
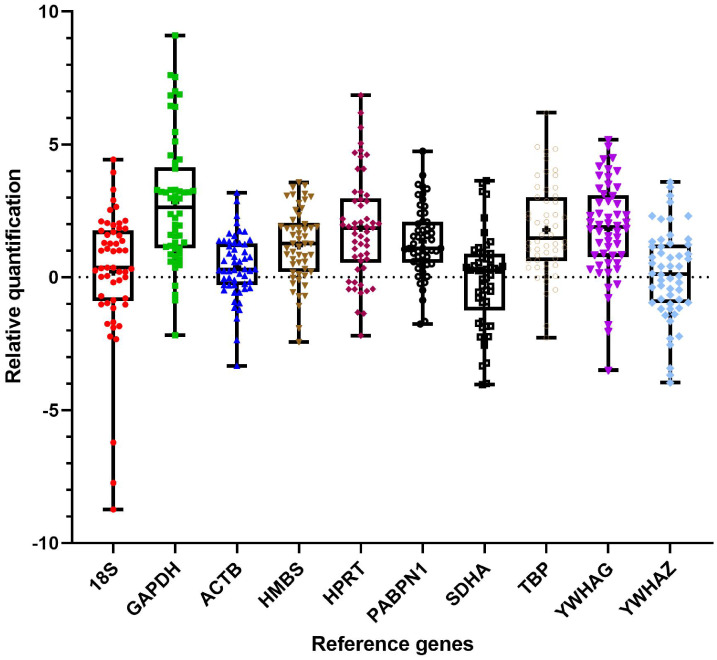
Relative quantitation of reference genes. ΔCq values for reference genes in a representative experimental run. Whiskers are 2.5% - 97.5% and ‘+’ denotes the arithmetic mean of Cq values for that reference gene.

Descriptive statistics of the gene expression of the candidate reference genes is in Table 2 and plate specific efficiencies and correction factors are listed below in Table 3. As expected *18S* showed the highest expression with an arithmetic and a geometric mean Cq of 15 while the lowest expression was *YWHAG*. The maximum standard deviation of Cq values was found for *18S*. *ACTB* and *PABPN1* had the lowest dispersion of Cq values from the mean. Reference gene Cq distributions were normal in each case and D’Agostino-Pearson test p > 0.05. A box plot of reference gene RQ values for a representative experimental run is presented in Fig 1. Amplification efficiency values were > 1.85 for all reference genes with the highest efficiency being 2. Mean efficiencies for the same reference gene were similar ±0.05 for all 3 experimental runs.

**Table 2:** Descriptive statistics of reference gene expression.

	18S	GAPDH	ACTB	HMBS	HPRT	PABPN1	SDHA	TBP	YWHAG	YWHAZ
**Number of values**	54	54	54	54	54	54	54	54	54	54
**Minimum Cq**	11.27	20.18	16.87	18.60	17.84	16.57	13.94	22.02	24.51	16.78
**Maximum Cq**	24.44	31.45	23.39	24.61	26.88	23.06	21.61	30.49	33.19	24.33
**Mean Cq**	15.49	26.45	19.67	20.97	22.82	19.96	17.72	26.43	27.87	20.25
**Std. deviation**	2.461	2.407	1.198	1.367	1.986	1.314	1.745	1.733	1.772	1.705
**Geometric mean**	15.32	26.34	19.64	20.92	22.73	19.92	17.63	26.37	27.81	20.18
**D’Agostino Pearson test K**^**2**^ **p-value**	0.08	0.16	0.13	0.54	0.43	0.71	0.82	0.73	0.17	0.74

Data shown for a representative experiment. Cq = quantification threshold; D’Agostino-Pearson test (Omnibus K2) p-value for distribution of *KIM-1* gene expression normalised to various normalisation factors. Null hypothesis for Omnibus K2 test = all values sampled from a Gaussian distribution.

**Table 3:** Correction factors, plate specific efficiencies and quantification threshold.

Gene	Correction factor (F_n_) Plate 1	Efficiency (E_n_) Plate 1	Nq Plate 1	Correction factor (F_n_) Plate 2	Efficiency (E_n_) Plate 2	Nq Plate 2
**18S**	0.94	1.88	41.00	1.06	1.86	20.68
**GAPDH**	1.41	2.00	44.30	0.71	2.00	52.59
**ACTB**	0.74	1.94	52.83	1.34	2.00	33.98
**HMBS**	0.7	1.97	69.18	1.43	1.92	52.32
**PABPN1**	0.97	1.84	60.42	1.03	1.85	107.28
**HPRT**	0.73	1.97	49.90	1.37	1.97	57.07
**SDHA**	0.66	1.98	48.45	1.51	1.87	32.84
**TBP**	0.88	2.00	66.59	1.14	2.00	63.73
**YWHAG**	0.33	2.00	17.64	2.00	2.00	33.12
**YWHAZ**	1.46	1.85	46.16	0.68	1.93	56.20

Data shown for a representative experiment.

### Reference gene stability

#### Reference gene ranking

Normfinder, qBase+, BestKeeper and comparative ΔCq approaches were used to assess the initial stability of candidate reference genes. The candidate reference genes are ranked in Table 4 with corresponding stability values in descending order with the most stable reference gene at the top. Normfinder ranked *HMBS* as the most stable (0.23) and *PABPN1* and *YWHAG* as second (0.36). For Normfinder, lower values have greater stability. For a 2 gene stability factor, Normfinder found *HMBS* and *YWHAG* as the best two genes to construct a normalisation factor of 0.187. The qBase+ algorithm ranked *ACTB*, *HMBS* and *PABPN1* as the top 3 most stable reference genes with respective ‘M’ values of 0.62, 0.69 and 0.71. A higher ‘M’ value denotes higher variation and less stability (see further evaluation of reference genes using qBase+ in S1 File). BestKeeper ranked *HMBS*, *YWHAG* and *PABPN1* as the most correlated genes in the constructed BestKeeper index and hence the most suitable stable reference genes. The standard deviations (± mean Cq) of the *HMBS*, *YWHAG* and *PABPN1* were 1.09, 1.36 and 1.09 respectively, all higher than the recommended standard deviation of 1 [26]. Only *ACTB* had a standard deviation (± mean Cq) less than 1 (0.92). The comparative ΔCq approach ranked *HMBS*, *PABPN1* and *ACTB* as the most stable. All techniques ranked *GAPDH* and *18S* as the least stable.

**Table 4:** Reference gene ranks.

Rank	Normfinder	qBase+ ^1^	BestKeeper ^2^	Comparative ΔCq ^3^
**1**	HMBS (0.26)	ACTB (0.62)	HMBS (0.94)	HMBS (1.23)
**2**	PABPN1 (0.36)	HMBS (0.69)	YWHAG (0.92)	PABPN1 (1.28)
**3**	YWHAG (0.36)	PABPN1 (0.71)	PABPN1 (0.88)	ACTB (1.33)
**4**	ACTB (0.42)	YWHAZ (0.83)	YWHAZ (0.88)	YWHAG (1.39)
**5**	YWHAZ (0.47)	YWHAG (0.9)	ACTB (0.86)	YWHAZ (1.46)
**6**	TBP (0.5)	TBP (1.0)	HPRT (0.82)	TBP (1.48)
**7**	HPRT (0.53)	HPRT (1.07)	TBP (0.81)	HPRT (1.57)
**8**	SDHA (0.71)	SDHA (1.22)	GAPDH (0.69)	SDHA (1.91)
**9**	18S (0.79)	GAPDH (1.36)	SDHA (0.63)	GAPDH (2.03)
**10**	GAPDH (0.85)	18S (1.56)	18S (0.57)	18S (2.52)

Ranking of reference genes from highest to lowest stability for each algorithm.

^1^geNorm (‘M’ value),

^2^Correlation coefficient ‘r’ to BestKeeper index,

^3^Average standard deviation.

As the different algorithms produced slightly differing rankings, we determined a consensus ranking using weighted rank aggregation. As there were 10 reference genes, we initially used the brute force approach with Spearman footrule distance applied to generate all possible rankings with minimum value of objective function. A second weighted consensus ranking was obtained with the cross-entropy Monte Carlo algorithm (in RStudio) (S2 File). Both brute force and cross-entropy Monte-Carlo approaches gave identical results (Table 5). *PABPN1* and *HMBS* were most stable using these methods, while *GAPDH* and *18S* were the least stable.

**Table 5:** Consensus ranking of reference genes.

Rank	Reference gene
**1**	*PABPN1*
**2**	*HMBS*
**3**	*ACTB*
**4**	*YWHAZ*
**5**	*YWHAG*
**6**	*TBP*
**7**	*HPRT*
**8**	*SDHA*
**9**	*GAPDH*
**10**	*18S*

Rank in descending order from most to least stable reference gene.

### 3-way linear mixed-effects model

Ideal normalisation factors for animal studies employ combinations of at least 2 reference genes [39]. ICC values ranged from 0 to 1, with higher values indicating greater stability. ICC estimates with a lower 95% CI limit less than 0.5, between 0.5 and 0.75, between 0.75 and 0.9, and greater than 0.90 are indicative of poor, moderate, good, and excellent reliability [40]. A normalisation factor constructed from *ACTB* and *PABPN1* was the most stable with an ICC of 0.86 (95% CI, 0.65–1), indicating moderate to good reliability. The best 3-reference gene normalisation factor was *ACTB*, *PABPN1* and *YWHAG* with an ICC of 0.77 (95% CI, 0.55–0.99), but with a lower limit less than *ACTB*×*PABPN1* (Table 6). Given the experimental conditions, a minimum sample size of 106 would have been required to improve the precision of the ICC estimate and to narrow the width of the 95% CI to a width of 0.1 (S3 File, section 3). S4 File in Table 1 in Excel^™^ S4 File represents a tabulation of minimal sample sizes necessary to arrive at two to five ‘true reference genes’ with ICC ranging between 0.7 and 0.9 and two-sided confidence interval width = 0.1 and 0.2. ‘True reference genes’ are the reference genes determined to be the most stable for the specific experiment instead of the candidate reference genes. For an example, in the present experiment, there are 10 candidate reference genes but only two ‘true reference genes’ as determined by the 3-way LMM method. The accompanying Excel^™^ calculator tool can also provide the researcher with the minimum sample sizes necessary for a desired ICC and confidence interval width.

**Table 6:** Top 3 most stable reference genes combinations using 3-way LMM.

Rank	Gene combination	ICC	LRT p-value	Width of confidence interval ‘w’
**1**	*ACTB* × PABPN1*	0.858	0.154	0.21
**2**	*ACTB* × *PABPN1* × *YWHAG*	0.778	0.073	0.22
**3**	*ACTB* × *YWHAG*	0.744	0.086	0.25

* Optimal reference gene combination with highest stability/reliability and no systemic variation by group. Reference gene combinations ranked in descending order of stability according to the 3-way linear mixed model. ICC = intraclass correlation coefficient; LRT p-value = Likelihood ratio test to determine if gene expression variation is due to systemic effects or their interaction with reference genes is significant. Group = treatment group. w: Confidence interval width ranks the ICC; a shorter width indicates greater accuracy. Since reference genes should be stable across groups without systemic effects, p-value for group/systematic effects (LRT p-value) for reference genes should be > 0.05.

#### Relevance of selecting a particular normalisation factor

Four commonly queried GOIs in the IRI and toxicological renal injury literature (*KIM-1*, *PECAM1*, *HIF1α* and *TGFβ1*) were analysed to test the impact of choice of normalisation factors/reference genes. Depending on reference gene/normalisation factor used, significant discrepancies in GOI fold differences between experimental groups were found in both ischaemic and toxicological injury models.

*KIM-1* is a transmembrane glycoprotein expressed in proximal tubules and upregulated following AKI. Its ectodomain appears in urine after cisplatin toxicity, IRI and after feeding 0.25% adenine [41, 42]. *KIM-1* was selected to demonstrate the effect of selection of reference genes on normalisation of GOI since its expression varies with different acute challenges. *PECAM1* is an endothelial cell junction molecule also expressed to different degrees on leukocyte sub-types and platelets [43]. Paralleling peritubular capillary rarefaction, *PECAM1* is reduced in kidney cortex and outer strip of outer medulla in rodent IRI models [44]. *HIF1α* is a master regulator of cell responses to hypoxia, expressed in both proximal and distal tubular cells and leads to expression of several genes involved in adaptation to decreased oxygen availability [45]. *TGFβ1* is a modulator of fibrosis in many models of tissue injury and is upregulated in proximal tubular epithelial cells after renal IRI [46].

**Fig 2:**
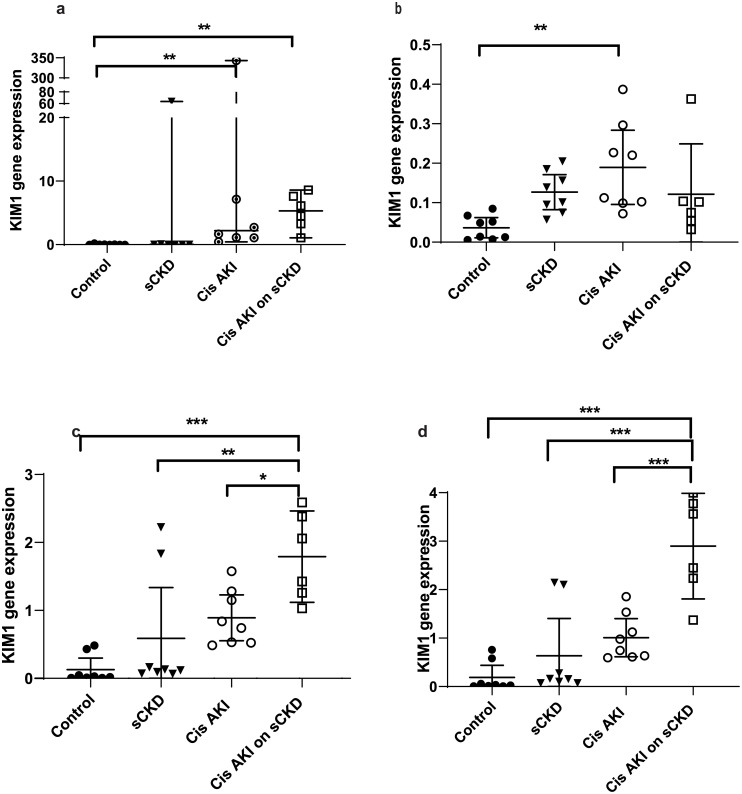
Expression of *KIM-1* normalised to a) *18S*, b) *GAPDH*, or c) *PABPN1×HMBS or d) ACTB×PABPN1* following AKI induced by Cisplatin or IRI. Data are means (one-way ANOVA) or medians (Kruskal Wallis) and 95% CI (n ≥ 6). * p < 0.05, ** p < 0.01, *** p < 0.001.

**Fig 3:**
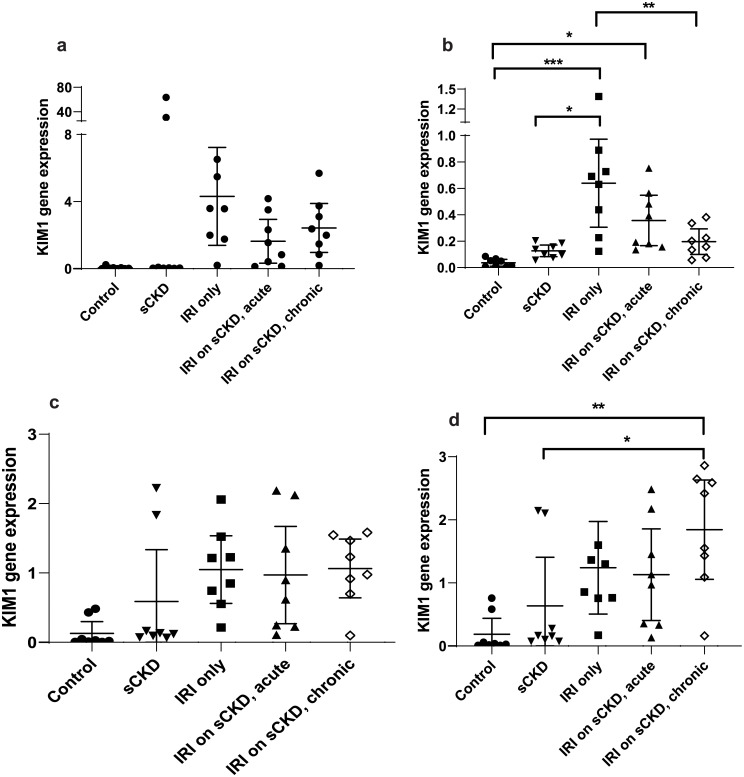
Expression of *KIM-1* normalised to a) *18S*, b) *GAPDH*, c) *PABPN1×HMBS*, d) *ACTB×PABPN1* in the IRI treated groups. Data are means (one-way ANOVA) or medians (Kruskal Wallis) and 95% CI (n ≥ 6). * p < 0.05, ** p < 0.01, *** p < 0.001.

**Fig 4:**
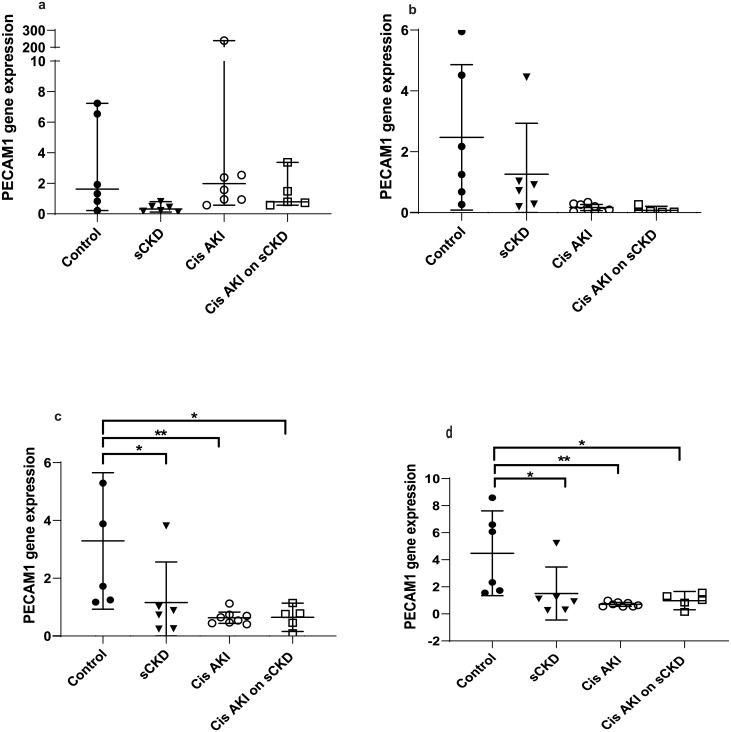
Expression of *PECAM1* normalised to a) *18S*, b) *GAPDH*, c) *PABPN1×HMBS*, d) *ACTB×PABPN1* in the cisplatin treated groups. Data are means (one-way ANOVA) or medians (Kruskal Wallis) and 95% CI (n ≥ 6). * p < 0.05, ** p < 0.01, *** p < 0.001.

**Fig 5:**
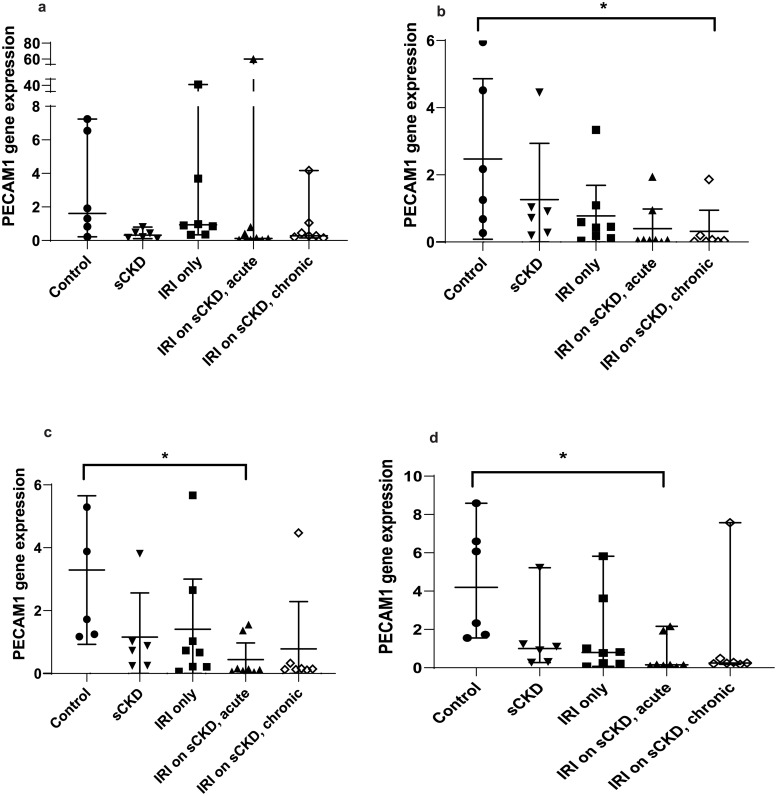
Expression of *PECAM1* normalised to a) *18S*, b) *GAPDH*, c) *PABPN1×HMBS*, d) *ACTB×PABPN1* in the IRI treated groups. Data are means (one-way ANOVA) or medians (Kruskal Wallis) and 95% CI (n ≥ 6). * p < 0.05, ** p < 0.01, *** p < 0.001.

**Fig 6:**
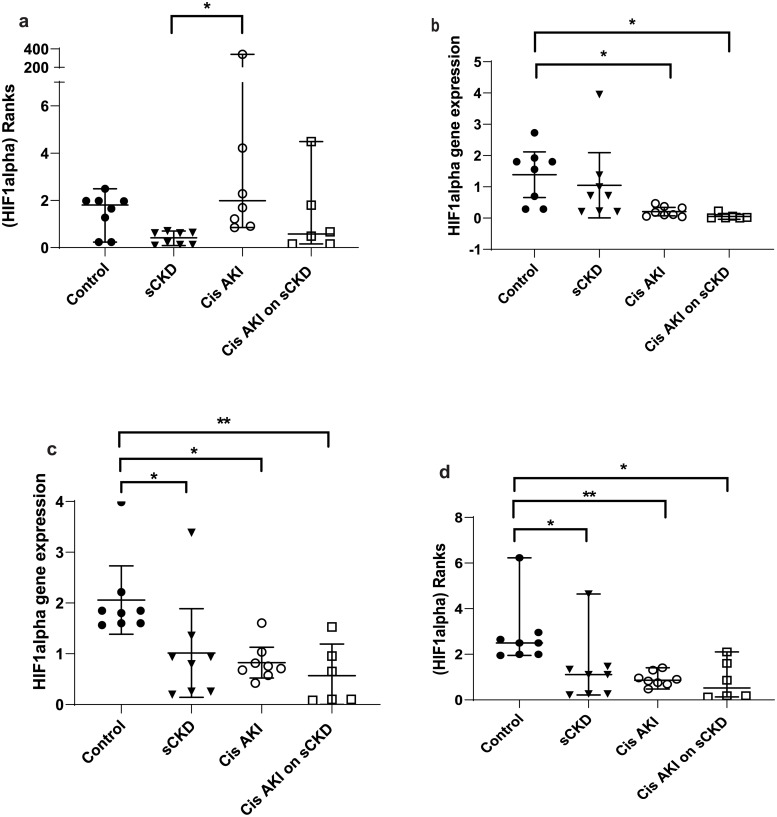
Expression or rank difference between groups of *HIF1α* normalised to a) *18S*, b) *GAPDH*, c) *PABPN1×HMBS*, d) *ACTB×PABPN1* in the cisplatin treated groups. Data are means (one-way ANOVA) or medians (Kruskal Wallis) and 95% CI (n ≥ 6). * p < 0.05, ** p < 0.01, *** p < 0.001.

**Fig 7:**
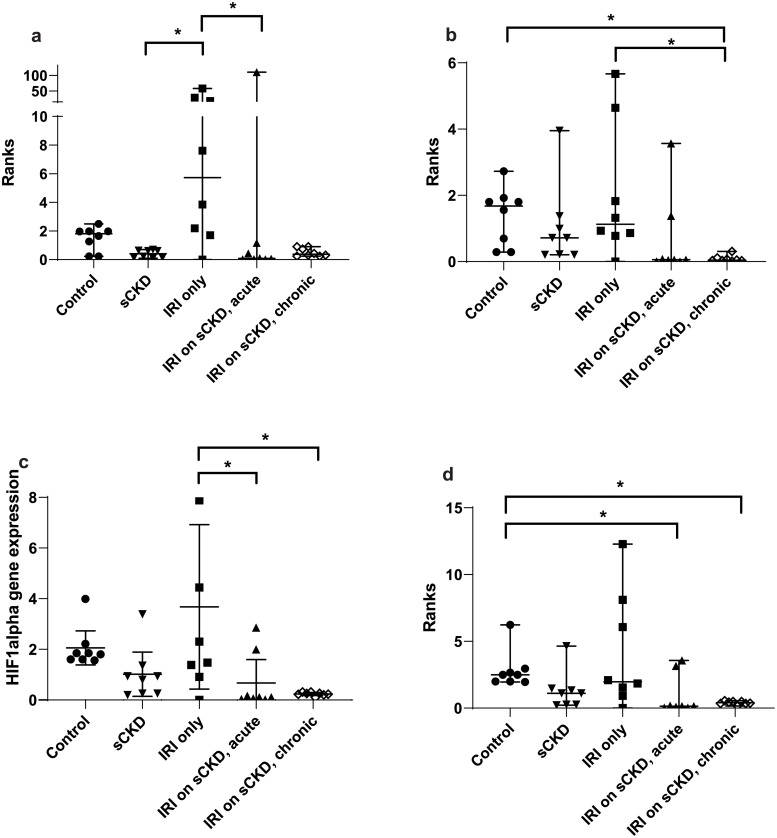
Expression or rank difference between groups of *HIF1α* after IRI and normalised to a) *18S*, b) *GAPDH*, c) *PABPN1×HMBS*, d) *ACTB×PABPN1*. Data are means (one-way ANOVA) or medians (Kruskal Wallis) and 95% CI (n ≥ 6). * p < 0.05, ** p < 0.01, *** p < 0.001.

**Fig 8:**
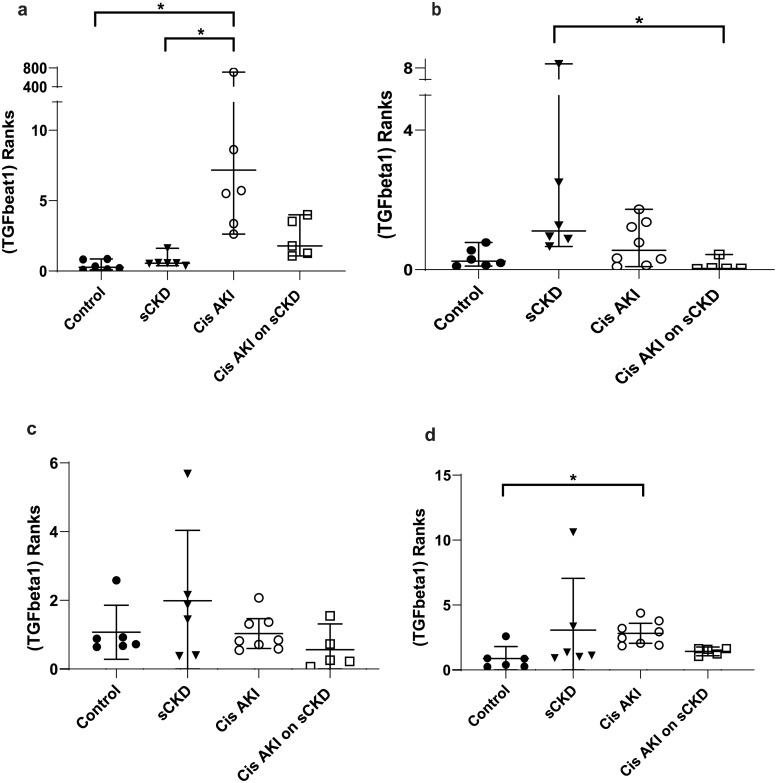
Rank difference between groups of *TGFβ1* expression after cisplatin and normalised to a) *18S*, b) *GAPDH*, c) *PABPN1×HMBS*, d) *ACTB×PABPN1*. Data are means (one-way ANOVA) or medians (Kruskal Wallis) and 95% CI (n ≥ 6). * p < 0.05, ** p < 0.01, *** p < 0.001.

**Fig 9:**
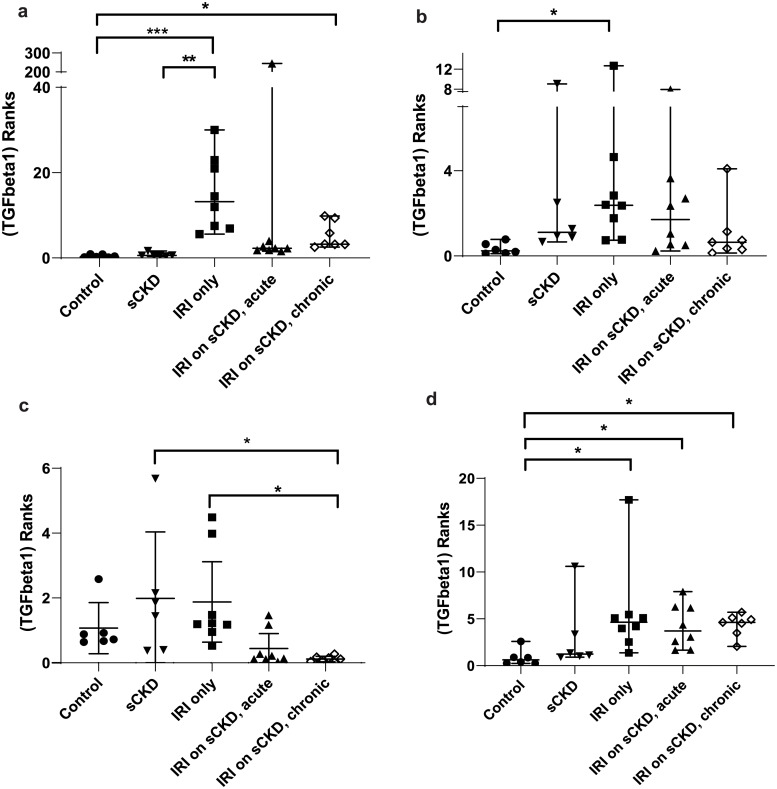
Rank difference between groups of *TGFβ1* expression after IRI and normalised to a) *18S*, b) *GAPDH*, c) *PABPN1×HMBS*, d) *ACTB×PABPN1*. Data are means (one-way ANOVA) or medians (Kruskal Wallis) and 95% CI (n ≥ 6). * p < 0.05, ** p < 0.01, *** p < 0.001.

GOI expression was assayed under the same conditions as the candidate reference genes and the normalised relative quotients (NRQs) were obtained by normalising the GOIs against reference genes/reference gene combinations (Figs 2–9). Tables 7, 9, 11 and 13 summarises the statistical methods used to analyse the respectively *KIM-1*, *PECAM1*, *HIF1α* and *TGFβ1* gene expression and Tables 8, 10, 12 and 14 summarises the significance of experimental treatment group comparisons of the respective GOIs, when normalised against 4 reference genes or reference gene combinations.

**Table 7:** Statistical methods used to analyse *KIM-1* gene expression.

		Normalisation factor			
GOI	Treatment	*18S*	*GAPDH*	*HMBS×PABPN1*	*ACTB×PABPN1*
***KIM-1***	Cisplatin	Kruskal-Wallis/ Dunn’s (Fig 2a)	One-way ANOVA/ Turkey’s (Fig 2b)	One-way ANOVA/ Turkey’s (Fig 2c)	One-way ANOVA/ Turkey’s (Fig 2d)
	IRI	One-way ANOVA/ Turkey’s (Fig 3a)	One-way ANOVA/ Turkey’s (Fig 3b)	One-way ANOVA/ Turkey’s (Fig 3c)	One-way ANOVA/ Turkey’s (Fig 3d)

Analysis of variance and post hoc multiple comparisons. Ordinary one-way analysis of variance (ANOVA) and Tukey’s multiple comparison tests were used for data with a Gaussian distribution. The Kruskal-Wallis and Dunn’s multiple comparisons were tests utilised to analyse data with a non-parametric distribution. Corresponding figure is in brackets. GOI = gene of interest

**Table 8:** Statistical differences of *KIM-1* normalised to different reference genes and reference gene combinations.

			Normalisation factor			
GOI	Treatment	Group Comparison	*18S*	*GAPDH*	*HMBS×PABPN1*	*ACTB×PABPN1*
***KIM-1***	Cisplatin	Control vs cis AKI only	p < 0.01	p < 0.01	NS	NS
		Control vs cis AKI on sCKD	p < 0.01	NS	p < 0.001	p < 0.001
		sCKD only vs cis AKI on SCKD	NS	NS	p < 0.01	p < 0.001
		Cis AKI only vs cis AKI on sCKD	NS	NS	p < 0.05	p < 0.001
	IRI	Control vs IRI only	NS	p < 0.001	NS	NS
		Control vs IRI on sCKD (acute)	NS	p < 0.01	NS	NS
		sCKD only vs IRI only	NS	p < 0.001	NS	NS
		IRI only vs IRI on sCKD (chronic)	NS	p < 0.01	NS	p < 0.01
		sCKD only vs IRI on sCKD (chronic)	NS	NS	NS	p < 0.05

**NS =** No significant difference

**Table 9:** Statistical methods used to analyse *PECAM1* gene expression.

		Normalisation factor			
GOI	Treatment	*18S*	*GAPDH*	*HMBS×PABPN1*	*ACTB×PABPN1*
***PECAM1***	Cisplatin	Kruskal-Wallis/ Dunn’s (Fig 4a)	One-way ANOVA/ Turkey’s (Fig 4b)	One-way ANOVA/ Turkey’s (Fig 4c)	One-way ANOVA/ Turkey’s (Fig 4d)
	IRI	Kruskal-Wallis/ Dunn’s (Fig 5a)	One-way ANOVA/ Turkey’s (Fig 5b)	One-way ANOVA/ Turkey’s (Fig 5c)	Kruskal-Wallis/ Dunn’s (Fig 5d)

Analysis of variance/ post hoc multiple comparisons; Ordinary one-way analysis of variance (ANOVA) and Turkey’s multiple comparisons test for data which has a Gaussian distribution. Kruskal-Wallis/ Dunn’s multiple comparisons utilised to analyse data with non-parametric distribution. Corresponding figure is in brackets. GOI = gene of interest

**Table 10:** Statistical differences of *PECAM1* normalised to different reference genes and reference gene combinations.

			Normalisation factor			
GOI	Treatment	Group Comparison	*18S*	*GAPDH*	*HMBS×PABPN1*	*ACTB×PABPN1*
***PECAM1***	Cisplatin	Control vs sCKD only	NS	NS	p < 0.05	p < 0.05
		Control vs cis AKI	NS	p < 0.05	p < 0.01	p < 0.01
		Control vs cis AKI on sCKD	NS	p < 0.05	p < 0.05	p < 0.05
		sCKD only vs cis AKI only	p < 0.01	NS	NS	NS
	IRI	Control vs IRI on sCKD (acute)	NS	NS	p < 0.05	p < 0.01
		Control vs IRI on sCKD (chronic)	NS	p < 0.05	NS	NS

NS = No significant difference

**Table 11:** Statistical methods used to analyse *HIF1α* gene expression.

		Normalisation factor			
GOI	Treatment	*18S*	*GAPDH*	*HMBS×PABPN1*	*ACTB×PABPN1*
***HIF1α***	Cisplatin	Kruskal-Wallis/ Dunn’s (Fig 6a)	One-way ANOVA/ Turkey’s (Fig 6b)	One-way ANOVA/ Turkey’s (Fig 6c)	Kruskal-Wallis/ Dunn’s (Fig 6d)
	IRI	Kruskal-Wallis/ Dunn’s (Fig 7a)	Kruskal-Wallis/ Dunn’s (Fig 7b)	One-way ANOVA/ Turkey’s (Fig 7c)	Kruskal-Wallis/ Dunn’s (Fig 7d)

Analysis of variance/ post hoc multiple comparisons; Ordinary one-way analysis of variance (ANOVA) and Turkey’s multiple comparisons test for data which has a Gaussian distribution. Kruskal-Wallis/ Dunn’s multiple comparisons utilised to analyse data with non-parametric distribution. Corresponding figure is in brackets. GOI = gene of interest

**Table 12:** Statistical differences of *HIF1α* normalised to different reference genes and reference gene combinations.

			Normalisation factor			
GOI	Treatment	Group comparison	*18S*	*GAPDH*	*HMBS×PABPN1*	*ACTB×PABPN1*
***HIF1α***	Cisplatin	Control vs sCKD only	NS	NS	p < 0.05	p < 0.05
		Control vs cis AKI	NS	p < 0.05	p < 0.05	p < 0.05
		Control vs cis AKI on sCKD	NS	p < 0.05	p < 0.01	p < 0.01
		sCKD only vs cis AKI only	p < 0.01	NS	NS	NS
	IRI	Control vs IRI on sCKD (acute)	NS	NS	NS	p < 0.05
		Control vs IRI on sCKD (chronic)	NS	p < 0.05	NS	p < 0.05
		sCKD only vs IRI only	p < 0.05	NS	NS	NS
		IRI only vs IRI on sCKD (acute)	p < 0.05	NS	p < 0.05	NS
		IRI only vs IRI on sCKD (chronic)	NS	p < 0.05	p < 0.01	NS

NS = No significant difference

**Table 13:** Statistical methods used to analyse *TGFβ1* gene expression.

		Normalisation factor			
GOI	Treatment	*18S*	*GAPDH*	*HMBS×PABPN1*	*ACTB×PABPN1*
***TGFβ1***	Cisplatin	Kruskal-Wallis/ Dunn’s (Fig 8a)	Kruskal-Wallis/ Dunn’s (Fig 8b)	One-way ANOVA/ Turkey’s (Fig 8c)	Kruskal-Wallis/ Dunn’s (Fig 8d)
	IRI	Kruskal-Wallis/ Dunn’s (Fig 9a)	Kruskal-Wallis/ Dunn’s (Fig 9b)	One-way ANOVA/ Turkey’s (Fig 9c)	Kruskal-Wallis/ Dunn’s (Fig 9d)

Analysis of variance/ post hoc multiple comparisons; Ordinary one-way analysis of variance (ANOVA) and Turkey’s multiple comparisons test for data which has a Gaussian distribution. Kruskal-Wallis/ Dunn’s multiple comparisons utilised to analyse data with non-parametric distribution. Corresponding figure is in brackets. GOI = gene of interest

**Table 14:** Statistical differences of *TGFβ1* normalised to different reference genes and reference gene combinations.

			Normalisation factor			
GOI	Treatment	Group Comparison	*18S*	*GAPDH*	*HMBS×PABPN1*	*ACTB×PABPN1*
***TGFβ1***	Cisplatin	Control vs cis AKI only	p < 0.001	NS	NS	p < 0.01
		sCKD only vs cis AKI only	p < 0.01	NS	NS	NS
		sCKD only vs cis AKI on sCKD	NS	p < 0.05	NS	NS
	IRI	Control vs IRI only	p < 0.001	p < 0.05	NS	p < 0.05
		Control vs IRI on sCKD (acute)	NS	NS	NS	p < 0.05
		Control vs IRI on sCKD (chronic)	p < 0.05	NS	NS	p < 0.05
		sCKD only vs IRI only	p < 0.001	NS	NS	NS
		sCKD only vs IRI on sCKD (chronic)	NS	NS	p < 0.05	NS
		IRI only vs IRI on sCKD (chronic)	NS	NS	p < 0.05	NS

NS = No significant difference

For cisplatin induced injury, normalisation of GOIs, *KIM-1*, *PECAM1*, *HIF1α* and *TGFβ1* against the 4 reference genes or reference gene combinations produced varying results (Figs 2, 4, 6 and 8 respectively). All GOIs normalised against *18S* or *GAPDH* produced discrepant results compared with normalisation against the more stable normalisation factors of *HMBS×PABPN1* and *ACTB×PABPN1*. However, as expected from stable normalisation factors, the pattern of fold differences between various experimental treatment groups were similar when *KIM-1*, *PECAM1* and *HIF1α* were normalised against *HMBS×PABPN1* (Figs 2c, 4c and 6c) or *ACTB×PABPN1* (Figs 2d, 4d and 6d). *KIM-1* and *PECAM1* gene expression patterns were more consistent with histological injury when normalised against the stable normalisation factors *HMBS×PABPN1* or *ACTB×PABPN1* compared to either *18S* or *GAPDH*.

For IRI, the fold expression of *KIM-1*, *PECAM1*, *HIF1α* and *TGFβ1* observed among treatment groups depended on the normalisation factor employed (Figs 3, 5, 7 and 9 respectively). As expected, *KIM1* and *TGFβ1* showed a pattern of upregulation with increasing injury when normalised against the stable *ACTB×PABPN1* (Figs 3d and 9d). *PECAM1* gene expression tended to decrease with severity of renal injury and normalisation against *GAPDH* (Fig 5b) along with more stable *HMBS×PABPN1* (Fig 5c) and *ACTB×PABPN1* (Fig 5d) produced this pattern. *HIF1α* which is induced by reperfusion and oxygen availability is downregulated 24 hours after insult [47]. Tissues from all treatment groups in the present study were obtained either on day 7 or 14 after cisplatin or IRI; this might explain the discrepant *HIF1α* results irrespective of reference gene or reference gene combination (Figs 6a–6d, 7a–7d).

2-way ANOVA analysis of *KIM-1*, *PECAM1*, *HIF-1α* and *TGFβ1* gene expression results (NRQs) confirmed superiority of using the geometric mean of 2 reference genes rather than one (p < 0.05) (S5 File, S5 Figs 1–4 in S5 file and S4 File in Table 1). For example, normalisation against *PABPN1* did not demonstrate a difference in gene expression between control and IRI on sCKD (acute) or IRI on sCKD (chronic) groups, in contrast to normalisation against the geometric mean of *ACTB*×*PABPN1*.

## Discussion

This is the first study to our knowledge that compares the most reliable reference genes for normalisation for RT-qPCR for transcript GOI levels in rat kidneys under ischaemic and toxicological conditions using multiple statistical approaches [48]. *HMBS* and *PABPN1* were among the top 3 most stable genes according to Normfinder, qBase+, BestKeeper and comparative ΔCq methods. In contrast, the most commonly used reference genes in rat studies, *18S* and *GAPDH*, were the least stable of the 10 genes assessed by all algorithms. The robustness of these results was highlighted by analysis using brute force and Monte Carlo cross entropy weighted aggregation that produced identical rankings. After weighted aggregation, *PABPN1*, *HMBS* and *ACTB* were ranked the most reliable reference genes. In contrast, in mouse models of renal cystic disease, *GAPDH*, *peptidylprolyl isomerase A* and *phosphoglycerate kinase* have been ranked highest, highlighting the need for model, species and strain specific housekeeping genes [31].

Reference gene selection was further refined and allowance made for experimental group variation not accounted for by the current models by development of a 3-way LMM. This model defined confidence intervals for stability values and group sizes and was superior in selecting optimal reference genes. In addition, it accommodated multiple continuous and categorical variables with sample random effects, gene fixed effects, systematic effects, and gene by systematic effect interaction. These are major advantages of using the 3-way LMM. Reference gene combinations can help reduce the measurement errors in single reference gene and improve reliability in the gene normalisation process, and the 3-way LMM provides statistical inference, including p-value and confidence intervals for stability. Statistical inference allows the selected reference gene combination to be generalizable to other experiments.

geNorm and its’ newer version qbase+, selects reference genes by using the standard deviation of respective gene expressions as a stability measure. It calculates the variation in the log-transformed gene expression ratio ‘M’, between a candidate reference gene with respect to all other reference genes in pairwise comparisons across all samples [14, 27]. A higher ‘M’ value represents greater variation in gene expression and less stability. qbase+ follows a step-down approach to remove genes with the highest M-value step by step and recalculates M-values for the remaining genes. There is no objective cut-off point to determine when to stop the process. This approach means that the algorithm tends to select the most correlated genes rather than the candidate reference genes with the least variable expression. qbase+ cannot take into account covariates such as systemic effects or interactions between genes and systematic effects in analysis.

To determine the optimal number of reference genes required to construct the most stable normalisation factor, qbase+ calculates the average variation between log-transformed expression ratios of sequential normalisation factors (NF_n_/NF_n+1_). The authors showed that a pairwise variation (NF_n_/NF_n+1_) < 0.15 is unlikely to be improved with the inclusion of additional reference genes [14]. The linear mixed model (LMM) searches for the optimum number and gene combinations to construct the most stable normalisation factor. The linear mixed model (LMM) algorithm stops if the lower bound of 95% confidence interval of ICC does not increase for higher-order gene combinations. Using the lower bound of 95% confidence interval for ICC takes the variation of the stability measure into account and avoids selection of genes with ICC estimated so imprecisely that the researcher cannot be confident of a high value.

Sample size calculation is based on the accuracy of the stability measure to aid optimal experimental design. The minimum effective sample size calculations take into account the number of reference genes studied, study design, stability level and desired confidence interval. The aim of the present study was to determine the most stable normalisation factor, i.e., the best combination of reference genes for a specific set of experimental conditions. GOIs will vary with experimental conditions and the correct sample size for the GOI must be determined independently. However, sample sizes in a gene expression study are dependent on both the effect size of the intervention (numerator) and stability of the reference genes utilised (denominator). The 3-way LMM, takes into account both continuous and categorical covariates and allows the minimal sample sizes required to determine this denominator with the least the error i.e. the most stable reference gene combination (normalisation factor) for a given experiment. As all statistical inferences were incorporated in the unified mixed-effects model, statistical inference for stability measures and systematic effects can be analyzed simultaneously. Furthermore, combining multiple statistical inferences in one model prevents inflation of Type I error. Calculation of the overall optimal sample size for a specific power (1-β) in a GOI experiment depends on the fold difference, i.e., the effect size of the specific GOI under the experimental conditions used (e.g., *KIM-1*) [49]. However, since GOI expression is shown as a ratio of the raw GOI data to the normalisation factor. Reduced error or variation in references genes reduces variation after GOI normalisation. Choosing a normalisation factor with an ICC of close to 0.9 (*ACTB×PABPN1*) increases the reliability of the group differences in GOI expression for a given experimental group size. Less reliable normalisation factors with lower ICC will have higher co-variates. If variance between groups is increased, larger experimental group sizes will be necessitated to maintain power and reliability of any experimental group differences.

This can be further demonstrated by the following; essentially, the researcher is looking at the variance of the quotient of GOI/reference gene combination. The formula to calculate the variance of a product is complex and shown below [50].

Here, X = GOI, Y = 1/reference gene combination, μ_x_ = mean of x and μ_y_ = is mean of Y.

If X and Y are not independent, the product of the variance is calculated as below:

$$\text{Var}\left(\text{X}.\text{Y}\right)=\text{Cov}\left({\text{X}}^{2},{\text{Y}}^{2}\right)+((\text{Var}\left(\text{X}\right)+\mu_{x}^{2}).(\text{Var}(\text{Y})+\mu_{y}^{2}))-(\text{Cov}(\text{X},\text{Y})-\mu_{\text{x}}\mu_{\text{y}}{)}^{2},$$

However, if covariance were zero and GOI and HKG are independent i.e. not varying with each other in some way then:

$$\text{Var}(\text{X}.\text{Y})=(\text{Var}(\text{X}).\text{Var}(\text{Y}))+(\text{Var}(\text{X}).\mu_{y}^{2})+(\text{Var}(\text{Y}).\mu_{x}^{2})$$

This value for Var(X.Y) is clearly going to be a greater value if there is any significant covariance. The lesser the ICC the greater the covariates hence a greater variance and standard deviation GOI/reference gene. The standard deviation of GOI/reference gene is used to calculate power and a reduced standard deviation results in potentially smaller group sizes required to detect difference between experimental groups.

Thus, it is clear that any covariance between the variables increases the variance of GOI/reference gene and hence a larger sample size will be required for the same level of significance in a power calculation for determination of significant differences in expression of GOIs between groups. However, calculation of a change in sample size based on the variance of the product of GOI and 1/reference gene is technically challenging unless the covariance values are known in advance for the experimental conditions under study.

Two further considerations are the relative abundance of reference gene expression and functional class. The most stable reference genes (*HMBS*, *PABPN1* and *ACTB*) had similar levels of expression but the Cq values were almost an average 5 cycles lower than less stable genes such as *GAPDH* and *TBP*. While lower abundance may theoretically increase error due to lower fluorescence intensity, this did not occur. *GAPDH* and *18S* were of higher abundance and ranked the lowest by all 4 algorithms. Identifying genes from different functional classes (*HMBS*, *PABPN1* and *ACTB)* reduces the risk of selecting co-regulated genes.

Normalisation by the geometric mean of at least 2 reference genes has been strongly advocated due to potential for confounding [4, 51, 52]. The data confirm that the geometric mean of 2 genes was superior to a single reference gene. Most modern PCR machines can run 2 fluorescence detection channels simultaneously using a probe-primer system. While additional probes are more expensive, a single reaction with reduced risk of technical and experimental variation may ultimately be more time and cost efficient.

There are limitations to this study. The pattern of injury is not homogenous throughout the cortex and medulla under light microscopy; kidney samples that included both unaffected cortex and medulla do not take account of heterogeneity of injury or cell type [53, 54]. Nevertheless, most gene expression studies in rat kidneys are performed on pooled tissue and use methodology consistent with ours. Consequently, the most stable reference genes in this study are likely to have external validity for toxic and ischaemic injury in rodent kidney. Note that protein expression does not necessarily correlate with gene expression for many reasons including variable effective translation and protein turnover [55–57].

Determination of gene expression stability requires evaluation within the context of treatment and tissue [10, 52]. Major practical considerations in performing assessments of the best reference genes include cost, animal usage and tissue of interest. Combining the data set for analysis in our studies likely resulted in higher expression variability of the reference genes. This was reflected in the high sample size required to minimise the 95% confidence interval of even the most stable normalisation factor and highlights problems with variation due to the effect of covariates even with the most stable reference genes when treatment groups are pooled for evaluation.

Species and intervention type may also be important considerations. No studies have assessed the biological variability of reference genes in kidneys of different rat strains. In liver, *18S* was the most stable reference gene for both Wistar and Zucker rats [38]. The SD rat strain was used in the present kidney studies. It is likely that both intervention and tissue type contribute to variability of gene expression. Cost and logistical difficulties likely limit academic laboratories from using multiple sets of reference genes to normalise RT-qPCR study data with identical animal species and developmental stages.

## Conclusion

The results emphasise the need to determine stable reference genes and a geometric mean of 2 stable reference genes is superior to 1 for normalising a GOI. *ACTB* and *PABPN1*, validated as stable under multiple experimental conditions, provided optimal stability as reference genes. The 3-way LMM provided an effective method for identifying stable pairs of reference genes in any context. These techniques should reduce variance and increase reproducibility and reliability of pre-clinical studies.

## Materials and methods

### Animals

Animal work was conducted in strict accordance with the Australian code for the care of animals for scientific purposes (National Health and Medical Research Council, 2013) and approved by the Animal Ethics Committee at the University of New South Wales (ACEC Approval 14\133A). All surgery was performed under isofluorane anaesthesia, and all efforts were made to minimise suffering. Rats were randomly allocated to the experimental treatment groups and subjected to nephrotoxic challenges (adenine or cisplatin) and/or ischaemia-reperfusion induced kidney injury as shown in Table 15 and S6 File (S6 Figs 1–3 in S6 File). Adverse events or deaths prior to endpoint were not observed. Animals were humanely euthanised under anaesthesia. The animal models, creatinine assays and histopathology are described in S6 and S7 Files [58].

**Table 15:** Experimental groups and interventions.

Group	Group no.	Abbreviation	No. rats	Intervention	Day of cull
Control	1	Control	8	Normal diet	63
Subclinical chronic kidney disease	2	sCKD	8	0.25% adenine diet for 4 weeks and 4 weeks of normal chow	56
Toxic acute kidney injury	3	Cis-AKI	8	Normal chow for 8 weeks, 4mg/kg cisplatin induced AKI on day 56	63
Toxic acute kidney injury on adenine induced chronic kidney disease	4	Cis-AKI on sCKD	6	0.25% adenine diet for 4 weeks, 4 weeks of normal chow, cisplatin 4mg/kg on day 56	63
Ischaemic acute kidney injury on subclinical chronic kidney disease, acute group	5	IRI on sCKD, (acute)	8	0.25% adenine diet for 4 weeks, 4 weeks of normal chow, 30min of bilateral renal ischaemia on day 56 then reperfusion < 24 hours	57
Ischaemic acute kidney injury on subclinical chronic kidney disease, chronic group	6	IRI on sCKD, (chronic)	8	0.25% adenine diet for 4 weeks, 4 weeks of normal chow, 30min of bilateral renal ischaemia on day 56 then recovery for 2 weeks	70
Ischaemic acute kidney injury only	7	IRI only	8	Normal chow for 8 weeks, 45 min of unilateral ischaemia on day 56, then nephrectomy of contralateral non-ischaemic kidney	56

AKI = acute kidney injury; Cis-AKI = Cisplatin induced acute kidney injury; IRI = ischaemia reperfusion injury; sCKD = subclinical chronic kidney disease

### Reference gene selection

Reference genes were selected from a literature search of rat studies (Table 16). Primers were designed using Beacon Designer (Palo Alto, California, USA). All primer pairs were intron-spanning, non-homologous to other rat genes and avoided structural folding areas.

**Table 16:** Reference genes and GOI primers.

Gene name (symbol)	Accession number	Forward primer (5’-3’)	Reverse primer (5’-3’)	Tm (°C)	Amplicon length	Ref
Ribosomal 18S (*18S*)	X01117	GATGCTCTTAGCTGAGTG	GTTCCGAAAACCAACAAA	60		[37]
Glyceraldehyde 3-phosphate dehydrogenase (*GAPDH*)	NM_017008	CTACCCACGGCAAGTTCAAC	CCAGTAGACTCCACGACATA	59.4	138	[12]
Beta-actin (*ACTB*)	V01217	AAGTCCCTCACCCTCCCAAAAG	AAGCAATGCTGTCACCTTCCC	61.3	75	[12]
Hydroxymethylbilane synthase (*HMBS*)	NM_013168	TCTAGATGGCTCAGATAGCATGCA	TGGACCATCTTCTTGCTGAACA	59.4	76	[12]
Polyadenylate-binding nuclear protein 1 (*PABPN1*)	NM_001135008	AGAGCGACATCATGGTAT	CATCAAGGTCATCTTCTGTT	59.4	127	[12]
Hypoxanthine-guanine phosphoribosyltransferase (*HPRT*)	NM_012583	TCATATCAGTAACAGCATCTAAG	GAACGGTTGACAACGATT	59.4	79	[9]
TATA binding protein (*TBP*)	NM_001004198	TGCTGGTGATTGTTGGTT	GGAAGGCGGAATGTATCTG	61.3	199	[2]
Succinyl dehydrogenase (*SDHA*)	NM_130428	AAGCACACCCTCTCATAT	CAGTCAGCCTCATTCAAG	59.4	92	[2]
14-3-3 protein gamma (*YWHAG*)	NM_019376	CAGTTCTCTATTTTGTTTTC	TCACTTGATTAGACCTTAA	56.9	196	[9]
Tyrosine 3-monooxygenase/ tryptophan 5-monooxygenase activation protein zeta (*YWHAZ*)	NM_013011.2	GATGAAGCCATTGCTGAACTTG	GTCTCCTTGGGTATCCGATGTC	56.9	117	[12]
Kidney injury molecule (*KIM-1*)	AF035963	GAAGATGTAGTCTCTGTCA	CATACTGGTTGGTTCCTA	59.4	N/A	
Platelet And Endothelial Cell Adhesion Molecule 1 (*PECAM1)*	NM_031591	GCTAACTTCACCATCCAGAA	CCTCTCCTCGGCAATCTT	61.4	75	[44]
Hypoxia Inducible Factor 1 Subunit Alpha (*HIF1α*)		CCTGCACTGAATCAAGAGGTGC	CCATCAGAAGGACTTGCTGGCT	59.4	175	[45]
Transforming Growth Factor Beta 1 (*TGFβ1*)	AY550025	AACCAAGGAGACGGAATA	GTGGAGTACATTATCTTTGCT	61.4	75	[46]

N/A = not applicable

### RT-qPCR

Specific RNA extraction and RT-qPCR methods are detailed in S8 File. Reference genes and *KIM-1* were amplified in triplicate in all samples per experimental run. Triplicates were re-assayed if there were missing values or if Cq differences were > 1 cycle within the triplicate. The maximum Cq value for reference genes was 34 cycles. Intra-experiment variation between technical replicates was < 1.2% and inter-experiment variation was < 1.5%. Samples were distributed over two 96-well plates in each experimental run and hence a factor correction for inter-plate variation was performed for each run [59]. Additional details of inter-plate variation correction are in S8 File. Plate specific amplification efficiencies were generally similar (± 0.05) for a given reference gene.

### Statistical analyses

For expression stability analysis, plate corrected triplicate averages (i.e., raw Cq values) or relative quantities (RQs) were used (S9 File). Expression stability was analysed using NormFinder, qBase+, BestKeeper and comparative ΔCq statistical algorithms to determine the most stable reference gene or gene pairs for normalisation. The 4 algorithms produced slightly varying results. Hence a universal rank was constructed by using brute force and Monte Carlo cross entropy methods (S2 File) utilising RankAggreg package in RStudio [60]. The workflow describing input data and expression stability analysis for these statistical algorithms is detailed in S8 File.

#### Linear mixed-effects model

To refine the reference gene selection process further, a 3-way LMM was developed that used the ICC of gene expression levels as the stability measure to rank reference genes with low residual variation within the intervention group and minimal between group variation. The 3-way LMM accommodated nested experimental designs, estimated variance components for determination of confidence intervals of stability values and provided minimum effective sample sizes for selection of reference genes for future studies [27].

The 3-way LMM was constructed with samples nested in experimental treatment groups. Systemic effects included gene expression variation due to experimental intervention and effects due to interactions with the reference genes. The reference gene combination with an ICC (*ρ)* with a 95% CI that has the highest lower limit is selected as the most stable normalisation factor. This provides an algorithm to determine the total sample size necessary to select reference genes with least uncertainty [28]. The minimum effective sample size necessary can be calculated to accurately estimate ICC of a given set of reference genes with desired precision (i.e. width of the [*100(1-α)*]% CI) [28, 61]. A detailed workflow of the 3-way LMM and the formulas for effective sample size calculation are described in S3 File. 3-way LMM calculations were performed using SAS software (version 9.4, 2017, SAS Institute Inc, Cary, North Carolina, USA).

#### Normalised relative quotients

NRQs for GOIs was calculated as described by Hellemans, et al [26] and this is detailed in S10 File, formulas 11–13. Normality of the NRQ data set for each GOI and reference gene/ reference gene combination was confirmed by D’Agostino-Pearson test. The mean amplification efficiency and RQs for all GOIs are listed in S11 File (S11 File, Table 1 in S11 File).

#### Further statistical analysis

Gene expression results are presented as NRQ and expressed as means ± standard deviations. Comparisons were made using 1-way or 2-way ordinary ANOVA and Tukey’s or Bonferroni’s multiple comparison tests (post hoc) when the distribution of the variables was normal. The Kruskal Wallis test statistic was calculated and Dunnett’s multiple comparison test was performed (post hoc) for non-normally distributed data. The D’Agostino-Pearson test was used to assess normality.

## Supporting information

S1 File qBase+, calculation of average pairwise variation. (DOCX)

S2 File RStudio code for Rankaggreg and BruteAggreg. (DOCX)

S3 File 3-way linear mixed model, SAS macro and sample size calculation for evaluation of ‘true reference genes’. (DOCX)

S4 File Minimal sample sizes needed in experiments to detect ‘true reference genes’. (XLS)

S5 File Differences in *GOI* gene expression when normalised to various normalisation factors. (DOCX)

S6 File Experimental rat groups, ischaemic and toxic injury protocol. (DOCX)

S7 File Methods for creatinine assay and histopathology. (DOCX)

S8 File RT-qPCR, correction of inter-plate variation, analytical methods and removal of outliers when constructing Bestkeeper index. (DOCX)

S9 File Relative gene expression quantities (RQ) of samples per each candidate reference gene. (DOCX)

S10 File Derivation of NRQ and corresponding standard errors. (DOCX)

S11 File RT-qPCR amplification efficiency and delta Cq results of GOIs. (DOCX)
