# Supplementary material for: Selection and validation of reference genes for normalisation of gene expression in ischaemic and toxicological studies in kidney disease
Source: PLoS One. 2020 May 21;15(5):e0233109. doi: 10.1371/journal.pone.0233109 (PMC7241806; doi:10.1371/journal.pone.0233109)
Supplement: S1 File — (DOCX) [file pone.0233109.s001.docx]

**Supplement 1**

**qbase+, calculation of average pairwise variation**

To determine the optimal number of reference genes required to construct the most stable normalisation factor, qbase+ calculates the average pairwise variation (V_n_/V_n+1_) between log_2_-transformed expression ratios of sequential normalisation factors (normalisation factor_n_ (NF_n_)/normalisation factor_n+1_ (NF_n+1_)). Authors of geNorm (precursor to qbase+), showed that a pairwise variation (V_n_/V_n+1_) < 0.15 was unlikely to be improved with additional reference genes (1, 2). A column graph of average variation of log_2_-transformed expression ratios of normalisation factors in descending order of stability is depicted in S7 Fig 1. In our study, troughs in this pairwise variation (V_n_/V_n+1_) never descend below the threshold of 0.15. However inclusion of a 3rd (0.164) or a 7^th^ gene (0.16) appear to offer the most stable normalisation factor. In this scenario, a compromise between practicality and experimental rigor is use of a 3 reference gene normalisation factor.

**Figure 1. Column graph of average variation of log_2_-transformed expression ratios of normalisation factors (NF) with decreasing order of stability (NF1 → NF10).**

**NF2 =** Normalisation factor constructed from the geometric mean of the 2 most stable reference genes. Hellamans and Vendesompele et al (2) showed that normalisation factor is unlikely to increase in stability once a threshold of 0.15 is reached

**References**

1. Vandesompele J, De Preter K, Pattyn F, Poppe B, Van Roy N, De Paepe A, et al. Accurate normalization of real-time quantitative RT-PCR data by geometric averaging of multiple internal control genes. Genome Biol. 2002;3(7):Research0034.
2. Jan Hellemans GM, Anne De Paepe, Frank Speleman and Jo Vandesompele. qBase relative quantification framework and software for management and automated analysis of real-time quantitative PCR data. Genome Biol. 2007.
