# Supplementary material for: Selection and validation of reference genes for normalisation of gene expression in ischaemic and toxicological studies in kidney disease
Source: PLoS One. 2020 May 21;15(5):e0233109. doi: 10.1371/journal.pone.0233109 (PMC7241806; doi:10.1371/journal.pone.0233109)
Supplement: S2 File — (DOCX) [file pone.0233109.s002.docx]

**Supplement 2**

**RStudio Code for Rankaggreg and BruteAggreg**

## RankAggreg package (1) (available through CRAN http://cran.r-project.org/web/ packages/RankAggreg) with RStudio employed to construct this consensus ranking.

## Data was entered into a Microsoft Excel™ spreadsheet, saved as a comma delineated file (.csv) and imported to ‘RStudio’ as described below. Then load ‘RankAggreg’ version 0.6.5.

data1 <- read.csv("dataset/HKG_rank_namecsv.csv")

data2 <- read.csv("dataset/HKG_rank_scrorecsv.csv")

### remove the 1st col from data 1

data1_modified <- data1[, -1]

### convert the data1_modified to a matrix

data1_modified <- as.matrix(data1_modified)

### remove the 1st col in data 2

data2_modified <- data2[, -1]

### convert the data2_modified to a matrix

data2_modified <- as.matrix(data2_modified)

library(RankAggreg)

toprank <- BruteAggreg(data1_modified, 10, weights = data2_modified, distance = "Spearman")

secondAlgorithm <- RankAggreg(data1_modified, 10, weights = data2_modified, method = "CE", distance = "Spearman")

**References**

1. Vasyl Pihur SDaSD. RankAggreg, an R package for weighted rank aggregation. BMC Bioinformatics. 2009;10(62).
