# Supplementary material for: Selection and validation of reference genes for normalisation of gene expression in ischaemic and toxicological studies in kidney disease
Source: PLoS One. 2020 May 21;15(5):e0233109. doi: 10.1371/journal.pone.0233109 (PMC7241806; doi:10.1371/journal.pone.0233109)
Supplement: S3 File — (DOCX) [file pone.0233109.s003.docx]

**Supplement 3**

**3-way linear mixed model**

Construction of the three-way LMM for selection and testing reference genes with the strongest intraclass correlation coefﬁcient (ICC) was presented by Dai et al (1) and would not repeated here. In our study, samples were nested within experimental treatment groups. Hence the systemic effect variance component was due to the experimental treatments and the sample random effects component accounted for by correlations among genes within the same experimental treatment group. The ICC could be described as the ratio between inter-experimental variation to the sum of inter-experimental group and intra-experimental group variation (residual variation), then maximising ICC is equivalent to minimising the residual variation when taking the sum of variations into account. Furthermore, ideal reference genes should be free of systemic main effects (β_1_) and systemic effects by gene interaction (β_2_). Therefore the null hypothesis can be described as, H_0_ : β_1,2 =_ $(\beta_{1}^{t},\beta_{2}^{t})^{t} = \overset{⇀}{0}$ and Hα : β_1,2_ ≠ $\overset{⇀}{0.}$Performing a global test with the likelihood ratio test (LRT) is used to evaluate H_0_ : β_1,2 =_  $\overset{⇀}{0}$and gene combinations with LRT < 0.05 are excluded from analysis.

**The application of the linear mixed model to our data set is forth with presented**

The linear mixed model (LMM) allows three types of effects: 1) Reference gene fixed effect, 2) sample random effect, and 3) systematic effect (in our study this is the treatment group variable only but the model allows multiple covariates e.g. age, other moderator group variables, weight etc). Sample random effects take the correlations among genes on the same sample into account.

Criteria: Ideal reference gene combination to construct the normalisation factor is the set of reference geness with the highest intraclass correlation coefficient (ICC) and no significant systemic effects (i.e. stable over treatment groups and other covariates with p-value>0.05).

1. **Data format**
   - Inter-plate calibration for same gene/experiment performed as described in supplements 2 and 3.
   - Then each efficiency corrected gene expression value (sample) is in the row.
   - Candidate reference genes are named as G1, G2, G3, and so on.
   - One or multiple systemic variables (Group, age, gender, race etc), either categorical or continuous, are allowed in the statistical modelling. In the present study, we only consider groups for systemic effects.
   - The LMM workflow as presented by Dai et al (1) is briefly;
     1. Start with exhaustive 2 gene combinations. Fit two genes G_1_ and G_2_ for $\forall$G_1_, G_2_ $\in$ (1,2……10 genes) to linear mixed model.
     2. Perform likelihood ratio test (LRT) to remove gene combinations with signiﬁcant systematic effects.
     3. ICC = $\text{ρ}_{G1G2} =\frac{\sigma_{\alpha}^{2}}{\left( \sigma_{\alpha}^{2} + \sigma_{\varepsilon}^{2} \right)}$ and 95% conﬁdence interval of ICC for genes G_1_ and G_2_. α_sample_ = between-subject variation and follows N(0,$\sigma_{\alpha}^{2})$while ε_sample, RG_ = sample variation and follows N(${0,\sigma}_{\varepsilon}^{2})$. ε_sample_,_RG_ and α_sample_ are mutually independent.
     4. Repeat Steps 1–3 for all 2 gene combinations within the 10 reference gene set. Similarly, steps 1-3 can be conducted for all 3-gene combinations, and so on. Usually, 2 to 3 gene combinations will be sufficient to serve as reference for normalization.
     5. In this study, all 2-gene and 3-gene combinations were considered in analysis. A gene combination with the highest lower bound of 95% conﬁdence interval of ICC and no signiﬁcant systematic effects (LRT p-value>0.05) is the optimal reference for normalization.

● Data for a single (i,e run 3) is presented in S5 table 1

**S3 Table 1. Data input for run 3**

| **G1** | **G2** | **G3** | **G4** | **G5** | **G6** | **G7** | **G8** | **G9** | **G10** | **Group** | **ID** |
| --- | --- | --- | --- | --- | --- | --- | --- | --- | --- | --- | --- |
| 6176 | 358827588 | 348777 | 709059 | 4188932 | 167492 | 22899 | 87340073 | 76366148 | 97680 | 1 | 1 |
| 2346 | 124551435 | 248483 | 364199 | 2107135 | 105147 | 12021 | 43508764 | 39575747 | 70207 | 1 | 2 |
| 3018 | 90392946 | 202180 | 338665 | 1169749 | 67871 | 12775 | 31672986 | 81612236 | 60275 | 1 | 3 |
| 3801 | 83383725 | 253260 | 451125 | 2293667 | 87724 | 9250 | 38661684 | 60412312 | 49344 | 1 | 4 |
| 1157 | 82519434 | 167773 | 253646 | 1168580 | 60475 | 9783 | 30161391 | 23923558 | 43567 | 1 | 5 |
| 15139 | 189098944 | 4017550 | 5169469 | 2649904 | 627148 | 884866 | 431487448 | 297588815 | 5340434 | 1 | 6 |
| 1561 | 67017483 | 1190209 | 2056242 | 5812427 | 250199 | 123732 | 122298422 | 627562063 | 905412 | 1 | 7 |
| 15826 | 505742915 | 1089283 | 2209829 | 1525586 | 255357 | 81670 | 241186432 | 763117991 | 653341 | 1 | 8 |
| 34917 | 7454699 | 114865 | 267110 | 858274 | 57283 | 1365804 | 19723402 | 162835849 | 326393 | 2 | 9 |
| 58558 | 5073217 | 93002 | 329997 | 179156 | 44194 | 1401633 | 19031044 | 117860659 | 389346 | 2 | 10 |
| 14943 | 293592504 | 7733782 | 1288151 | 4681465 | 1359670 | 826012 | 151308068 | 354867013 | 4431161 | 2 | 11 |
| 5170 | 810536693 | 2312486 | 2579009 | 2118178 | 455248 | 135828 | 479087841 | 768996477 | 1031218 | 2 | 12 |
| 5421 | 411028133 | 685100 | 1838597 | 8979633 | 250380 | 63260 | 241633152 | 241457998 | 255306 | 2 | 13 |
| 3531 | 308100129 | 422748 | 1097425 | 1099576 | 185325 | 55572 | 176017716 | 205845621 | 266182 | 2 | 14 |
| 5198 | 214930079 | 541604 | 1222831 | 1217793 | 180439 | 61092 | 130914670 | 181785622 | 805211 | 2 | 15 |
| 4869 | 286068283 | 485811 | 1433156 | 1462425 | 241704 | 123825 | 199646201 | 483925046 | 717222 | 2 | 16 |
| 35230 | 264474290 | 1512026 | 3697447 | 1173254 | 281225 | 331489 | 318153844 | 102492129 | 1220166 | 3 | 17 |
| 74578 | 374823725 | 1754392 | 4039269 | 2084799 | 454806 | 296596 | 303180666 | 691589884 | 893624 | 3 | 18 |
| 20801 | 66650328 | 778962 | 1721686 | 5257008 | 194517 | 311517 | 122435863 | 172618610 | 785275 | 3 | 19 |
| 16198 | 295632636 | 1570536 | 3122497 | 2529174 | 378223 | 180630 | 216295399 | 169592936 | 908288 | 3 | 20 |
| 53168 | 18847956 | 905755 | 1612401 | 4554484 | 157161 | 342562 | 67313150 | 223359882 | 1055118 | 3 | 21 |
| 33973 | 70990999 | 870904 | 2715318 | 5298695 | 289923 | 453385 | 150065076 | 479883120 | 1744381 | 3 | 22 |
| 2343697 | 26958426 | 990157 | 2249311 | 2545305 | 177364 | 432687 | 65165819 | 463914828 | 2144661 | 3 | 23 |
| 4395643 | 168220830 | 996035 | 4070153 | 7946696 | 361252 | 532443 | 249391299 | 112437756 | 3756941 | 3 | 24 |
| 5877 | 70367301 | 398823 | 715737 | 7493655 | 157298 | 140660 | 103213672 | 97748601 | 263356 | 4 | 25 |
| 18468 | 5658851 | 366610 | 472650 | 731889 | 68937 | 100971 | 13996842 | 53521047 | 171807 | 4 | 26 |
| 6390 | 1186196 | 281392 | 236001 | 278671 | 25495 | 84140 | 4252352 | 38244273 | 105106 | 4 | 27 |
| 8258 | 3483815 | 655834 | 737364 | 611461 | 90108 | 83208 | 10347055 | 122648674 | 238782 | 4 | 28 |
| 16289 | 7663561 | 440844 | 737364 | 2662826 | 144027 | 83432 | 21736750 | 47276978 | 172349 | 4 | 29 |
| 4974 | 5501444 | 336606 | 318159 | 773184 | 106246 | 94122 | 11756518 | 50979990 | 194510 | 4 | 30 |
| 5816 | 66218558 | 311692 | 766657 | 2982064 | 135534 | 41401 | 73182873 | 84548762 | 98039 | 5 | 31 |
| 10033 | 473304357 | 1126559 | 1442636 | 2256607 | 522518 | 78131 | 226635781 | 303191951 | 399530 | 5 | 32 |
| 6932 | 30108490 | 477493 | 1442636 | 4692927 | 214136 | 75931 | 37571002 | 259645905 | 456578 | 5 | 33 |
| 6808 | 305498271 | 688059 | 1175963 | 2496611 | 411249 | 53823 | 153674248 | 361551397 | 437706 | 5 | 34 |
| 8336 | 137090263 | 549107 | 1090411 | 8289278 | 253495 | 77914 | 127377015 | 209825007 | 328258 | 5 | 35 |
| 14773 | 71211609 | 318661 | 873104 | 2666833 | 128447 | 54783 | 81371992 | 102817533 | 243874 | 5 | 36 |
| 8405 | 350741190 | 1061109 | 1344636 | 2526104 | 536533 | 69269 | 190017122 | 595249802 | 574752 | 5 | 37 |
| 905878 | 102746096 | 1195699 | 2417961 | 4539617 | 782821 | 75039 | 303338434 | 593031400 | 558889 | 5 | 38 |
| 17953 | 442646548 | 735714 | 845723 | 2777681 | 341228 | 47973 | 150399782 | 334267706 | 274944 | 6 | 39 |
| 7858 | 123109444 | 677579 | 749050 | 4711837 | 327913 | 80024 | 136871799 | 166783533 | 181642 | 6 | 40 |
| 9489 | 3319079 | 323502 | 314998 | 816485 | 77077 | 65018 | 10922699 | 63104786 | 204866 | 6 | 41 |
| 9812 | 78599326 | 980822 | 673496 | 6111490 | 345363 | 165585 | 59088921 | 699439744 | 780365 | 6 | 42 |
| 9814 | 14604952 | 337932 | 394421 | 2105905 | 129888 | 55874 | 21765051 | 127153657 | 303248 | 6 | 43 |
| 9894 | 32763750 | 534385 | 604491 | 4641179 | 237109 | 50549 | 29498106 | 217128841 | 329579 | 6 | 44 |
| 30288 | 69173279 | 669368 | 981953 | 6525104 | 318424 | 84631 | 61117284 | 349763559 | 483891 | 6 | 45 |
| 28848 | 38218223 | 840752 | 1088081 | 5085295 | 323607 | 154258 | 74680407 | 261337080 | 190173 | 6 | 46 |
| 18294 | 68097546 | 265817 | 738648 | 404778 | 54563 | 33188 | 350486468 | 28277003 | 105600 | 7 | 47 |
| 14790 | 262713325 | 821946 | 2357128 | 5136505 | 239973 | 184645 | 163385168 | 485800334 | 411727 | 7 | 48 |
| 38185 | 164436277 | 659037 | 911675 | 4892790 | 182336 | 143008 | 102792615 | 245138647 | 205490 | 7 | 49 |
| 31598 | 70570706 | 318257 | 540129 | 4178635 | 152926 | 44925 | 61231664 | 203975550 | 154713 | 7 | 50 |
| 79280 | 222490445 | 502139 | 1501785 | 1066470 | 266092 | 103832 | 143370211 | 293084634 | 541932 | 7 | 51 |
| 19783 | 218954150 | 688794 | 1754805 | 7196626 | 288917 | 205983 | 200732224 | 389353374 | 521710 | 7 | 52 |
| 60541 | 430551935 | 957574 | 2966972 | 1019343 | 237786 | 334054 | 42477525 | 147758498 | 727971 | 7 | 53 |
| 55384 | 117589635 | 1862201 | 9095371 | 8244400 | 1283489 | 431619 | 110720058 | 978321782 | 1855966 | 7 | 54 |

G1 = 18S, G2 = GAPDH, G3 = ACTB, G4 = HMBS, G5 = HPRT, G 6 = PABPN1, G7 = SDHA, G8 = TBP, G9 = YWHAG and G10 = YWHAZ ; Group = treatment group, ID = sample.

1. **SAS macro were run (S3 table 2)**

Step One: Two SAS files (ICC_HKG SAS Macro.sas and ICC_HKG set-up.sas) were saved to PC.

Step Two: ICC_HKG set-up.sas program was modified to accommodate the data set of the present study.

**S3 Table 2**

| %let datalib= c:\experiment3; | Subdirectory for the existing input dataset |
| --- | --- |
| %let datain= Data3; | name of the existing input dataset. |
| %let method=ml; | "ml" or "reml". This defines the estimation method for parameters in mixed-effects models. |
| %let log="TRUE"; | "TRUE" will use natural log to transform the data. "FALSE" will analyze the raw data without log transformation |
| %let FourGenes="FALSE"; | "TRUE" will calculate ICC for four-gene combinations. Default ("FALSE) will calculate ICC for two-gene and three genes combinations. |
| %let id=ID; | Defines sample ID variable. |
| %let moderatorclass= group; | Categorical systematic variables (treatment group). |
| %let moderator=group; | Systematic effect or gene by systematic effect interaction. |
| %let n_gene=%eval(10); | Define the number of candidate genes in the data set. |

No changes were needed in the ICC-HKG SAS Macro.sas program file.

Step Three: Modified ICC-HKG set-up.sas program was run.

By default, the program considers all two-gene and three-gene combinations. Gene combinations with systematic effects p<0.05 are not suitable for housekeeping genes and thus they were removed. The remaining gene combinations were sorted by ICC.

The optimal reference gene is the one with the highest ICC and no systematic effects.

- ICC: Intraclass correlation coefficient (ranges between 0 and 1, higher values indicate stronger consistency/stability).
- LRT_pvalue: likelihood ratio test p-values for systematic effects (treatment group and other moderator effects). Since reference geness are expected to be stable over moderator (covariate) variables, gene combinations with p-values<0.05 have systematic effects and they are removed.

1. **Sample size calculation for evaluation of ‘true reference genes’**

A normalisation factor constructed from two or more ‘true’ reference genes minimises experimental error when analysing effect size of GOI. If the reference genes used to construct the normalisation factor are free of (or have minimal) systemic effects by gene interaction, the minimum effective sample size necessary to accurately estimate ICC (*ρ*) of a given set of ‘true’ reference genes with desire precision (i.e width of the [*100(1-α)*]% CI) can be calculated (1, 2).

The following formula provide power and sample size calculation for experiments to identify reference genes using three-way LMM.

Ideal reference genes for normalization do not have systematic effects or systematic effect by gene interactions.

Let m = be the number of true reference genes with known stability values for an experiment. For instance, if a 2-gene combination is used to build a normalisation factor then, m=2.

Let ρ = intra-class correlation coefficient of the set of reference genes ‘m’ above

Let *w* = be the required width of the confidence interval for ICC (stability) of the selected normalisation factor.

Then the minimum effective sample size (n) necessary to obtain a desired ‘*w*’ of CI of a ‘ρ’ for given combination of true reference genes is;

$n =\left[ \frac{8z_{\frac{\alpha}{2}}^{2}\left( 1-\rho\right)^{2} \times\left( 1+\left( m-1 \right)\rho\right)^{2}}{\left( mw^{2}\left( m-1 \right) \right)} \right]+1$ (1)

where $z_{\frac{\alpha}{2}}$= z score of an alpha in a two tailed distribution, eg. 1.96 for a 5% alpha level.

Derivation of formula (1) has been explained in section 2.4 by Dai et al (1) and will not be reproduced here.

Applying the data from the present study for a 2-reference gene normalisation factor and a 95% confidence interval;

$n = 8 \times\left( 1.96 \right)^{2} \left[ \frac{8 \times\left( 1.96 \right)^{2}\left( 1-0.86 \right)^{2} \times\left( 1+\left( 2-1 \right)0.86 \right)^{2}}{\left( 2w^{2} \left( 2-1 \right) \right)} \right] + 1$

Minimum total sample size ‘n’ required to obtain a confidence interval width, ‘w’ of 0.2 would be 26 and the required ‘n’ would be 106 to obtain a ‘w’ of 0.1.

**References**

1. Hongying Dai RC, Carrie A. Vyhlidal, Bridgette L. Jonesc and Madhusudan Bhandaryd. Mixed modeling and sample size calculations for identifying housekeeping genes. Statistitics in Medicine 2013 Published online (Wiley online library)
2. Bonett DG. Sample size requirements for estimating intraclass correlations with desired precision. Statistics in medicine. 2002;21(9):1331-5.
