# Supplementary material for: Selection and validation of reference genes for normalisation of gene expression in ischaemic and toxicological studies in kidney disease
Source: PLoS One. 2020 May 21;15(5):e0233109. doi: 10.1371/journal.pone.0233109 (PMC7241806; doi:10.1371/journal.pone.0233109)
Supplement: S5 File — (DOCX) [file pone.0233109.s005.docx]

**Supplement 5**

**Differences in GOI gene expression when normalised to various normalisation factors**

***KIM-1* gene expression normalised to either 1 reference gene (*PABPN1*) or the geometric mean of 2 reference genes (*ACTB×PABPN1*)**

**S5 Figure 1.** Multiple comparison of *KIM-1* NRQ against either normalisation factor constructed from a single gene, *PABPN1*, or normalisation factor of *PABPN1×ACTB*. Bars are means ± SD of the experimental intervention groups. 2-way ordinary ANOVA comparing means across intervention groups and normalisation factors. Statistically significant difference in main effects of normalisation factor and intervention in overall data sets between *KIM-1/PABPN1* vs *KIM-1/PABPN1×ACTB*, p < 0.005.

**S5 Table 1. 2-way ordinary ANOVA applied to data set of *KIM-1* gene expression**

**normalised against either *PABPN1* or geometric mean of *PABPN1*×*ACTB*.**

| **Source of variation** | **% of total variation** | **p- value** | **Significant?** |
| --- | --- | --- | --- |
| Treatment | 45.65 | <0.0001 | Yes |
| Normalisation factor | 5.473 | 0.0012 | Yes |
| **ANOVA table** | **DF** | **MS** | **P value** |
| Treatment | 6 | 7.582 | p < 0.0001 |
| Normalisation factor | 1 | 5.455 | p = 0.0012 |
| Residual | 94 | 0.4893 |  |

DF = the degrees of freedom in the source, MS = mean squares.

***PECAM1* gene expression normalised to either 1 reference gene (*PABPN1*) or the geometric mean of 2 reference genes (*ACTB×PABPN1*)**

**S5 Figure 2**. Multiple comparison of *PECAM1* NRQ against either normalisation factor constructed from a single gene, *PABPN1*, or normalisation factor of *PABPN1×ACTB*. Bars are means ± SD of the experimental intervention groups. 2- way ordinary ANOVA comparing means across intervention groups and normalisation factors. Statistically significant difference in main effects of normalisation factor and intervention in overall data sets between *HIF1α/PABPN1* vs *HIF1α/PABPN1×ACTB*, p < 0.001.

**S5 Table 2. 2-way ordinary ANOVA applied to data set of *PECAM1* gene expression**

**normalised against either *PABPN1* or geometric mean of *PABPN1*×*ACTB*.**

| **Source of variation** | **% of total variation** | **p- value** | **Significant?** |
| --- | --- | --- | --- |
| Treatment | 26.75 | 0.03 | Yes |
| Normalisation factor | 1.1 | 0.001 | Yes |
| **ANOVA table** | **DF** | **MS** | **P value** |
| Treatment | 6 | 15.31 | p < 0.03 |
| Normalisation factor | 1 | 3.66 | p < 0.001 |
| Residual | 41 | 0.13 |  |

DF = the degrees of freedom in the source, MS = mean squares.

***HIF1α* gene expression normalised to either 1 reference gene (*PABPN1*) or the geometric mean of 2 reference genes (*ACTB×PABPN1*)**

**S5 Figure 3.** Multiple comparison of *HIF1α* NRQ against either normalisation factor constructed from a single gene, *PABPN1*, or normalisation factor of *PABPN1×ACTB*. Bars are means ± SD of the experimental intervention groups. 2- way ordinary ANOVA comparing means across intervention groups and normalisation factors. Statistically significant difference in main effects of normalisation factor and intervention in overall data sets between *HIF1α/PABPN1* vs *HIF1α/PABPN1×ACTB*, p < 0.0001.

**S5 Table 3. 2-way ordinary ANOVA applied to data set of *HIF1α* gene expression normalised against either *PABPN1* or geometric mean of *PABPN1*×*ACTB***

| **Source of variation** | **% of total variation** | **p- value** | **Significant?** |
| --- | --- | --- | --- |
| Treatment | 31.24 | 0.01 | Yes |
| Normalisation factor | 0.77 | < 0.0001 | Yes |
| **ANOVA table** | **DF** | **MS** | **P value** |
| Treatment | 6 | 23.43 | p < 0.01 |
| Normalisation factor | 1 | 3.45 | p < 0.0001 |
| Residual | 41 | 0.14 |  |

DF = the degrees of freedom in the source, MS = mean squares.

***TGFβ1* gene expression normalised to either 1 reference gene (*PABPN1*) or the geometric mean of 2 reference genes (*ACTB×PABPN1*)**

**S5 Figure 4.** Multiple comparison of *TGFβ1* NRQ against either normalisation factor constructed from a single gene, *PABPN1*, or normalisation factor of *PABPN1×ACTB*. Bars are means ± SD of the experimental intervention groups. 2- way ordinary ANOVA comparing means across intervention groups and normalisation factors. Statistically significant difference in main effects of normalisation factor and intervention in overall data sets between *TGFβ1/PABPN1* vs *TGFβ1/PABPN1×ACTB*, p < 0.0001.

**S5 Table 4. 2-way ordinary ANOVA applied to data set of *TGFβ1* gene expression**

**normalised against either *PABPN1* or geometric mean of *PABPN1*×*ACTB*.**

| **Source of variation** | **% of total variation** | **p- value** | **Significant?** |
| --- | --- | --- | --- |
| Treatment | 11.93 | 0.04 | Yes |
| Normalisation factor | 19.43 | < 0.0001 | Yes |
| **ANOVA table** | **DF** | **MS** | **P value** |
| Treatment | 6 | 12.28 | p < 0.04 |
| Normalisation factor | 1 | 120 | p < 0.0001 |
| Residual | 41 | 3.29 |  |

DF = the degrees of freedom in the source, MS = mean squares.
