# Supplementary material for: Selection and validation of reference genes for normalisation of gene expression in ischaemic and toxicological studies in kidney disease
Source: PLoS One. 2020 May 21;15(5):e0233109. doi: 10.1371/journal.pone.0233109 (PMC7241806; doi:10.1371/journal.pone.0233109)
Supplement: S6 File — (DOCX) [file pone.0233109.s006.docx]

**Supplement 6**

**Experimental rat groups, ischaemic and toxic injury protocol**

**Methods**

**Animals**

All tissue samples were from male SD rats weighing approximately 250-300g, 6-8 weeks old at the start of the experiment and maintained at the Biological Resource Centre at the University of New South Wales. Rats were housed 3-4 animals per cage, acclimatised in a room maintained at 20°C and exposed to 12hour light dark cycle. All animals had free access to water. Rats received standard chow (Harlan Laboratories, Madison, Wisconsin) or chow supplemented with 0.25% adenine (Sigma Aldrich, Castle Hill, Australia) diets for four weeks; both diets were followed by standard chow for a further 4 weeks (total 8 weeks). Ischaemia-reperfusion injury was induced in selected groups at day 56. Blood was obtained from saphenous vein.

**Subclinical Chronic Kidney Disease.**

As previously described (1), adenine supplemented rats developed subclinical chronic kidney disease (sCKD), defined as renal histological injury developing without a significant change in renal function as measured by serum creatinine (and hence eGFR). S1 figure 1 describes overlay of ischaemia-reperfusion injury and cisplatin insult on adenine supplemented sCKD rats.

**Ischaemic Injury protocol**

Rats were initially anaesthetised with 3-4 % isoflurane and oxygen and then maintained at 1-2% isoflurane with oxygen. Buprenorphine was given for analgesia. Following bilateral loin incisions bilateral renal ischemia was induced by application of non-traumatic microvascular clamps around both left and right renal pedicles for either 30 minutes in adenine fed animals or 45 minutes for rats on a standard diet. The clamp duration was determined from pilot experiments that confirmed that 45 minutes of ischaemia in a normal kidney caused acute kidney injury (AKI) with increased serum creatinine and significant histological damage that recovered by 4 weeks post insult (1). Same experiments showed that 30 minutes of IRI in normal rats resulted in little or no change in serum creatinine 24 h post injury and minimal histological injury. To induce AKI in rats with underlying sCKD, rats received only 30 min of ischaemia followed by renal reperfusion. Rat tissue was obtained after IRI at the time of culling.

**Toxic injury protocol**

Acute cisplatin nephrotoxicity was induced by administration of cisplatin (4mg/kg) intraperitoneally, to both standard chow (group 3) and adenine fed sCKD rats (group 4). Tissues were obtained on day 7 post-cisplatin injury.

S1 figure 2 depicts rat treatment groups on standard chow who had either had 45minute of ischaemia and reperfusion injury or cisplatin insult on day 56. Control group which had rats on standard chow without any treatments or insults is also described in S1 figure 2. A summary of the various rat experimental groups, experimental group number and number of rats N per group is detailed in S1 figure 3.

**Induction of sCKD, IRI and cisplatin injury**

**
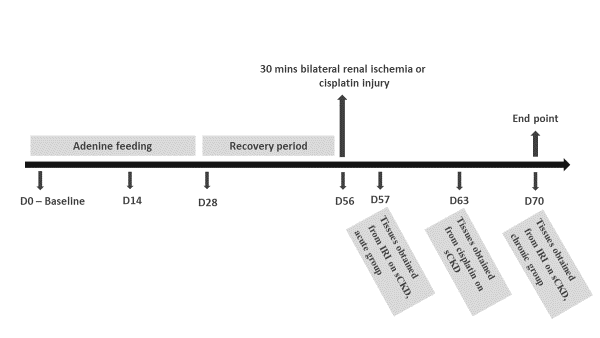
**

Tissues from IRI on on sCKD, chronic group

Tissues from IRI on on sCKD, acute group

Tissues from Cis-AKI on sCKD group

**S6 Figure 1.** Induction of sCKD and overlaying of IRI and Cisplatin injury (groups 2,4,5,6)

**Induction of IRI and cisplatin injury on normal diet**

**
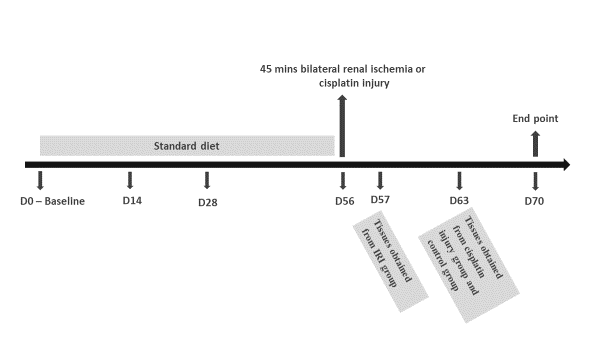
**

Tissues from Cis AKI and control groups

Tissues from IRI group

**S6 Figure 2.** Control, IRI and cisplatin groups on a standard chow (groups 1,3,7)

**Experimental rat groups**

N = 54

**IRI on sCKD**

**Control (group 1) N = 8**

**sCKD**

**(group 2)**

**N =8**

**Cis AKI**

**(group 3)**

**N =8**

**Cis AKI on sCKD**

**(group 4)**

**N = 6**

**Acute group**

**(group 5)**

**N = 8**

**Chronic group**

**(group = 6)**

**N = 8**

**IRI only**

**(group = 7)**

**N = 8**

**S6 Figure 3.** Various interventions, the respective group number and number of rats, N per group.

**References**

1. Succar L, Pianta TJ, Davidson T, Pickering JW, Endre ZH. Subclinical chronic kidney disease modifies the diagnosis of experimental acute kidney injury. Kidney international. 2017;92(3):680-92.
