# Supplementary material for: Selection and validation of reference genes for normalisation of gene expression in ischaemic and toxicological studies in kidney disease
Source: PLoS One. 2020 May 21;15(5):e0233109. doi: 10.1371/journal.pone.0233109 (PMC7241806; doi:10.1371/journal.pone.0233109)
Supplement: S7 File — (DOCX) [file pone.0233109.s007.docx]

**Supplement 7**

**Methods**

**Creatinine analysis**

Terminal serum creatinine was measured enzymatically using Konelab 630 automated analyser (Thermofisher, Waltham, MA) (57).

**Histopathology**

Micoronal slices (5mm) were immersion fixed in 10% formalin at room temperature. Sections fixed in formalin were dehydrated in graded alcohol (Tissue Tek VIP 4 Tissue processor, Sakura, Olympus, Australia) and embedded in paraffin. Haemotoxylin and eosin stained sections were examined using Aperio slide scanner and Image scope analysis software (Aperio, Vista, CA) at × 200 magnification for tuberlointerstitial disease. Tuberlointerstitial disease was scored (1 = < 25%, 2 = 26% - 50%, 3 = 51% -75%, 4 = 76% - 100%) per section and imaged by Aperio slide scanner at × 200 magnification to quantify cortical area. (57).
