# Supplementary material for: Selection and validation of reference genes for normalisation of gene expression in ischaemic and toxicological studies in kidney disease
Source: PLoS One. 2020 May 21;15(5):e0233109. doi: 10.1371/journal.pone.0233109 (PMC7241806; doi:10.1371/journal.pone.0233109)
Supplement: S8 File — (DOCX) [file pone.0233109.s008.docx]

**Supplement 8**

**RT-qPCR, correction of inter-plate variation, analytical methods and removal of outliers when constructing bestkeeper index**

**RNA extraction and cDNA synthesis**

Tissue was homogenised in Safe-Lock tubes (Eppendorf, Sigma Aldrich, USA) in a Bullet Blender (Gold NextAdvance NY, USA) for 4 minutes at a setting of 10. Total RNA was isolated using TRIsure (Bioline, London, UK) according to the manufacturer’s protocol. Sample purity was assessed by measuring OD A260/280 and values of 1-8-2.0 were acceptable. cDNA was synthesised from 1µg of total RNA using the Tetro cDNA reverse transcription kit (Bioline, London, UK) as per manufacturer. All primers were optimised to ensure specificity by melt curve analysis and amplification efficiency.

**RT-qPCR**

RT-qPCR was performed using Sensifast SYBR No-Rox mastermix (Bioline Meridean Bioscience, UK) as per manufacturer’s protocol. qPCR assays were spread over two 96-well plates per gene per experiment to accommodate all the treatment groups and technical replicates. Correction for inter-plate variation is described below**.**  Amplification efficiency for each plate was calculated with 5-serial 10-fold dilutions of standards. Standards were a mix of cDNA from 2 unique equal volume rat samples from each interventional group (hence total of 14 unique samples) and assayed in triplicate on each plate. Cq values vs log10 of diluted standard series were graphed and efficiency determined from log-linear part of the slope: Efficiency = 10^(-1/slope)^ for each 96-well plate (1). Only Cq values < 40 cycles were used to ensure that reference genes with reasonable expression or robust levels are used in the evaluation process.

**‘Correction of inter-plate variation for the same gene and experiment’ as described by Ruijter et al. (2)**

Correction of inter-plate variation for the same gene and experiment as described by Ruijter et al (2) who also is the author of ‘factor correction qPCR software (www.hartfaalcentrum.nl) that was used in this study. Maximum conditional overlap was achieved by spreading biological replicates across both plates and assaying identical standards on all plates. The technical triplicate values were then averaged for further analysis, once corrected Cq values had been obtained. All values depicted here for demonstrative purposes are from experimental run 3. Initially Cq values were converted to gene expression quantities (N_0_):

$N_{0} = \frac{N_{q}}{E^{Cq}}$ (1)

For each plate, a quantification threshold (Nq) value of relative fluorescence units was chosen, corresponding to the start of maximum amplification. Nq values of the same candidate reference gene were different between the two plates reflecting the random and technical variation between plates. E is plate specific efficiency value and Cq is the quantification cycle prior to plate correction.

Since our study had 54 unique samples, the biological error would be normally distributed with mean 0 and error σ satisfying the assumptions made in Ruijters et al model for calculating inter-plate variation. Run factor for run ‘n’ (F_n_) for each plate can now be calculated by using N_0_ values of standards and biological replicate samples that enabled maximum overlap of conditions between the two plates and which completed the between-run ratio matrix. F_n_ (‘n’ was either 1 or 2 as samples of the same gene were spread over only two plates) was calculated by using ‘factor correction_qPCR’ software and utilizes the concept:

Between run ratio_1/2_ = $\frac{Y_{1j}}{Y_{2j}}$ = $\frac{F_{1}}{F_{2}}$ $\times$ $\frac{\left( \overline{Y} + C_{j} + error \right)}{\left( \overline{Y} + C_{j}+ error \right)}$ , (2)

where ‘1’ and ‘2’ are the 1^st^ and 2^nd^ plates of the same gene in same experiment. Y_1j_ and Y_2j_ are the combined observations for plates 1 and 2. $\overline{Y}$ is the population mean, effect of condition ‘j’ is C_j_ and ‘error’ is the sum of technical errors for that particular plate.

Then corrected N_0_ (r_N_0_) for all the samples were calculated by dividing each sample by the plate specific correction factor (F_n_) obtained from above:

r_ N_0_ = $\frac{N_{0,n}}{F_{n}}$ (3)

An efficiency corrected Cq value (Cq_E,corr_) was calculated by

$Cq_{E,corr} = \frac{\log\left( 1 \right) - \log\left( r_{-}N_{0} \right)}{\log\left( E_{mean} \right)},$ (4)

whereas E_mean_, is the combined qPCR efficiency (i,e mean efficiency) for the 2-plate experiment per candidate reference gene (G) and was calculated by the formula:

$Emean,G=\frac{\sum_{N=1}^{2} \left( \mathrm{KnEn} \right)}{\sum_{N=1}^{2} Kn}$ (5)

where n is the number of plates per gene per experiment (n = 2 in our study), Kn is the number of observations for the specific gene on plate ‘n’ (Kn = 27 in our study), E_n_ is the reported efficiency for the specific plate ‘n’ and ‘G’ is the particular candidate reference gene.

Residual variation SSres was then calculated by:

SSres = $\overset{2}{}=\sum_{n=1}^{2} \left( \left( K_{n}-1 \right)\left( K_{n}\times\sqrt{SE_{n}} \right)^{2} \right)$, (6)

where SE_n_ is the combined standard error for plate ‘n’. Then standard error of the mean efficiency (SE_Emean)_ for the two plates was calculated by the formula:

$SE_{Emean, G} = \sqrt{\frac{SSres}{\frac{\sum\left( K_{n}-1 \right)}{\sum Kn}}}$ (7)

**Analytical methods**

Plate corrected technical triplicate averages were used to analyse expression stability by the Bestkeeper and comparative delta Cq algorithms. When employing Normfinder and qbase+, the technical triplicate values were first transformed into efficiency-corrected relative gene expression quantities (RQ) as described Pfaffl et al (1) using Microsoft Excel™. The workflow for determining RQs is shown in S6 formulas 8-10. The derived RQs for all samples/reference gene in experimental run 3 are listed in S3 Table 1.

The data was then converted and processed by each algorithm: NormFinder Excel add-on (Aarhus University Hospital, Denmark), qbase+ software, version 3.0 (Biogazelle, Zwijnaarde, Belgium - www.qbaseplus.com), Bestkeeper (Technical University of Munich, Germany) and the comparative deltaCq algorithm as described by Thein et al. (3). When constructing the Bestkeeper index, samples deviating by more than 3-fold (above or under expression) from the average Cq were removed as explained by Anderson et al (4) and is also described below. No outliers were removed when employing other algorithms to determine reference gene stability.

The results from the four statistical algorithms were used to construct an aggregate rank using both the brute force and Monte Carlo methods. The variability measurements from NormFinder, stability value ‘M’ obtained from qbase+, the Pearson correlation coefficients of the candidate reference genes to the Bestkeeper index from Bestkeeper approach and the average gene specific standard deviation from the comparative deltaCq approach were used as weights in the aggregation process. Both the brute force method and the cross-entropy Monte Carlo algorithm was used to arrive at the consensus rank as described by Pihur, et al. (5). Spearman footrule distance was used when utilising brute force method to find the consensus rank. Rank aggregations by both the brute force and the cross-entropy Monte-Carlo method was conducted with ‘RStudio Team (2015), RStudio: Integrated Development for R. RStudio, Inc., Boston, MA URL http://www.rstudio.com/’. The specific ‘RStudio’ code that was used in our study is listed in supplement 4.

**Removal of outliers when constructing Bestkeeper index**

Outliers are removed when constructing the Bestkeeper index (1) as it is assumed that they represent samples with poor RNA integrity and incomplete reverse transcription. Bestkeeper uses sample variance which is termed ‘InVar_m_ [±Cq]’ for gene ‘m’ to determine outliers.

Hence:

$InVar_{m}\left[ \pm Cq \right] = \frac{\sum_{i = 1}^{54} \left( Cq_{i}^{m}-avereageCq_{54} \right)}{53}$ (13)

where ‘m’ is the respective gene and ‘i’ is respective sample. Then ‘efficiency corrected x-fold variance’ in the sample set for a particular gene ‘m’ is calculated by utilizing ‘InVar_m_’ and the calculated mean qPCR amplification efficiency value for gene ‘m’:

$InVar_{m}\left[ \pm x_{-}fold \right] =E_{m}^{InVar\left[ \pm Cq \right]}$ (14)

Any sample value that deviates greater than 3-fold from the average Cq for the particular gene ‘m’ is removed from further analysis and construction of the Bestkeeper index.

**References**

1. Pfaffl MW. A new mathematical model for relative quantification in real-time RT-PCR. Nucleic acids research. 2001;29(9):e45-e.
2. Ruijter JM, Thygesen HH, Schoneveld OJLM, Das AT, Berkhout B, Lamers WH. Factor correction as a tool to eliminate between-session variation in replicate experiments: application to molecular biology and retrovirology. Retrovirology. 2006;3:2-.
3. Silver N, Best S, Jiang J, Thein SL. Selection of housekeeping genes for gene expression studies in human reticulocytes using real-time PCR. BMC molecular biology. 2006;7:33.
4. Andersen CL, Jensen JL, Orntoft TF. Normalization of real-time quantitative reverse transcription-PCR data: a model-based variance estimation approach to identify genes suited for normalization, applied to bladder and colon cancer data sets. Cancer Res. 2004;64(15):5245-50.
5. Vasyl Pihur SDaSD. RankAggreg, an R package for weighted rank aggregation. BMC Bioinformatics. 2009;10(62).
