# Supplementary material for: Selection and validation of reference genes for normalisation of gene expression in ischaemic and toxicological studies in kidney disease
Source: PLoS One. 2020 May 21;15(5):e0233109. doi: 10.1371/journal.pone.0233109 (PMC7241806; doi:10.1371/journal.pone.0233109)
Supplement: S9 File — (DOCX) [file pone.0233109.s009.docx]

**Supplement 9**

**Relative gene expression quantities (RQ) of samples per each candidate reference gene**

**S9 Table 1.**

| **Sample** | **Group** | **18S** | **GAPDH** | **ACTB** | **HMBS** | **HPRT** | **PABPN1** | **SDHA** | **TBP** | **YWHAG** | **YWHAZ** |
| --- | --- | --- | --- | --- | --- | --- | --- | --- | --- | --- | --- |
| 1 | 1 | 2.99 | 1.81 | 2.31 | 3.60 | 4.44 | 2.77 | 4.34 | 3.56 | 11.32 | 4.36 |
| 2 | 1 | 7.88 | 5.22 | 3.24 | 7.03 | 8.82 | 4.41 | 8.27 | 7.15 | 21.84 | 6.06 |
| 3 | 1 | 6.13 | 7.19 | 3.99 | 7.56 | 15.89 | 6.83 | 7.78 | 9.82 | 10.59 | 7.06 |
| 4 | 1 | 4.87 | 7.79 | 3.18 | 5.67 | 8.10 | 5.29 | 10.75 | 8.04 | 14.31 | 8.63 |
| 5 | 1 | 15.98 | 7.87 | 4.80 | 10.10 | 15.90 | 7.67 | 10.16 | 10.31 | 36.13 | 9.77 |
| 6 | 1 | 1.22 | 3.44 | 0.20 | 0.49 | 0.70 | 0.74 | 0.11 | 0.72 | 0.29 | 0.08 |
| 7 | 1 | 11.84 | 9.69 | 0.68 | 1.24 | 3.20 | 1.85 | 0.80 | 2.54 | 1.38 | 0.47 |
| 8 | 1 | 1.17 | 1.28 | 0.74 | 1.15 | 1.22 | 1.82 | 1.22 | 1.29 | 1.13 | 0.65 |
| 9 | 2 | 0.53 | 87.14 | 7.02 | 9.59 | 21.65 | 8.09 | 0.07 | 15.77 | 5.31 | 1.30 |
| 10 | 2 | 0.32 | 128.04 | 8.67 | 7.76 | 103.73 | 10.49 | 0.07 | 16.34 | 7.33 | 1.09 |
| 11 | 2 | 1.24 | 0.22 | 0.10 | 0.20 | 0.40 | 0.34 | 0.12 | 0.21 | 0.24 | 0.10 |
| 12 | 2 | 3.58 | 0.80 | 0.35 | 0.98 | 0.88 | 1.02 | 0.73 | 0.65 | 1.12 | 0.41 |
| 13 | 2 | 3.41 | 1.58 | 1.18 | 1.38 | 2.07 | 1.85 | 1.57 | 1.29 | 3.58 | 1.67 |
| 14 | 2 | 5.24 | 2.11 | 1.91 | 2.32 | 1.69 | 2.50 | 1.79 | 1.77 | 4.20 | 1.60 |
| 15 | 2 | 3.56 | 3.02 | 1.49 | 2.08 | 1.53 | 2.57 | 1.63 | 2.38 | 4.75 | 0.53 |
| 16 | 2 | 3.80 | 2.27 | 1.66 | 1.78 | 1.27 | 1.92 | 0.80 | 1.56 | 1.79 | 0.59 |
| 17 | 3 | 0.52 | 2.46 | 0.53 | 0.69 | 1.58 | 1.65 | 0.30 | 0.98 | 0.84 | 0.35 |
| 18 | 3 | 0.25 | 1.73 | 0.46 | 0.63 | 0.89 | 1.02 | 0.34 | 1.03 | 1.25 | 0.48 |
| 19 | 3 | 0.89 | 9.75 | 1.03 | 1.48 | 3.54 | 2.38 | 0.32 | 2.54 | 5.01 | 0.54 |
| 20 | 3 | 1.14 | 2.20 | 0.51 | 0.81 | 0.73 | 1.23 | 0.55 | 1.44 | 5.10 | 0.47 |
| 21 | 3 | 0.35 | 34.46 | 0.89 | 1.58 | 4.08 | 2.95 | 0.29 | 4.62 | 3.87 | 0.40 |
| 22 | 3 | 0.54 | 9.15 | 0.93 | 0.94 | 3.51 | 1.60 | 0.22 | 2.07 | 1.80 | 0.24 |
| 23 | 3 | 0.01 | 24.10 | 0.81 | 1.13 | 7.30 | 2.61 | 0.23 | 4.77 | 1.86 | 0.20 |
| 24 | 3 | 0.00 | 3.86 | 0.81 | 0.62 | 2.34 | 1.28 | 0.19 | 1.25 | 0.77 | 0.11 |
| 25 | 4 | 3.15 | 9.23 | 2.02 | 3.57 | 2.48 | 2.95 | 0.71 | 3.01 | 8.84 | 1.62 |
| 26 | 4 | 1.00 | 114.79 | 2.20 | 5.41 | 25.39 | 6.73 | 0.98 | 22.22 | 16.15 | 2.48 |
| 27 | 4 | 2.89 | 547.61 | 2.86 | 10.86 | 66.69 | 18.19 | 1.18 | 73.13 | 22.60 | 4.05 |
| 28 | 4 | 2.24 | 186.45 | 1.23 | 3.46 | 30.39 | 5.15 | 1.19 | 30.05 | 7.05 | 1.78 |
| 29 | 4 | 1.14 | 84.76 | 1.83 | 3.46 | 6.98 | 3.22 | 1.19 | 14.31 | 18.28 | 2.47 |
| 30 | 4 | 3.72 | 118.07 | 2.39 | 8.05 | 24.04 | 4.36 | 1.06 | 26.45 | 16.95 | 2.19 |
| 31 | 5 | 3.18 | 9.81 | 2.59 | 3.33 | 6.23 | 3.42 | 2.40 | 4.25 | 10.22 | 4.34 |
| 32 | 5 | 1.84 | 1.37 | 0.72 | 1.76 | 0.82 | 0.89 | 1.27 | 1.37 | 2.85 | 1.07 |
| 33 | 5 | 2.67 | 21.57 | 1.69 | 1.76 | 3.96 | 2.17 | 1.31 | 8.28 | 3.33 | 0.93 |
| 34 | 5 | 2.72 | 2.13 | 1.17 | 2.17 | 0.74 | 1.13 | 1.85 | 2.02 | 2.39 | 0.97 |
| 35 | 5 | 2.22 | 4.74 | 1.47 | 2.34 | 2.24 | 1.83 | 1.28 | 2.44 | 4.12 | 1.30 |
| 36 | 5 | 1.25 | 9.12 | 2.53 | 2.92 | 6.97 | 3.61 | 1.81 | 3.82 | 8.41 | 1.75 |
| 37 | 5 | 2.20 | 1.85 | 0.76 | 1.89 | 0.74 | 0.86 | 1.44 | 1.64 | 1.45 | 0.74 |
| 38 | 5 | 0.02 | 0.63 | 0.67 | 1.05 | 0.41 | 0.59 | 1.32 | 1.03 | 1.46 | 0.76 |
| 39 | 6 | 1.03 | 1.47 | 1.10 | 3.02 | 0.67 | 1.36 | 2.07 | 2.07 | 2.59 | 1.55 |
| 40 | 6 | 2.35 | 5.28 | 1.19 | 3.41 | 3.94 | 1.41 | 1.24 | 2.27 | 5.18 | 2.34 |
| 41 | 6 | 1.95 | 195.71 | 2.49 | 8.13 | 22.76 | 6.02 | 1.53 | 28.47 | 13.70 | 2.08 |
| 42 | 6 | 1.88 | 8.26 | 0.82 | 3.79 | 3.04 | 1.34 | 0.60 | 5.26 | 1.24 | 0.55 |
| 43 | 6 | 1.88 | 44.48 | 2.38 | 6.49 | 8.82 | 3.57 | 1.78 | 14.29 | 6.80 | 1.40 |
| 44 | 6 | 1.87 | 19.83 | 1.51 | 4.22 | 4.00 | 1.96 | 1.97 | 10.54 | 3.98 | 1.29 |
| 45 | 6 | 0.61 | 9.39 | 1.20 | 2.60 | 2.85 | 1.46 | 1.17 | 5.09 | 2.47 | 0.88 |
| 46 | 6 | 0.64 | 17.00 | 0.96 | 2.34 | 3.65 | 1.43 | 0.64 | 4.16 | 3.31 | 2.24 |
| 47 | 7 | 1.01 | 9.54 | 3.03 | 3.45 | 45.91 | 8.50 | 3.00 | 0.89 | 30.57 | 4.03 |
| 48 | 7 | 1.25 | 2.47 | 0.98 | 1.08 | 3.62 | 1.93 | 0.54 | 1.90 | 1.78 | 1.03 |
| 49 | 7 | 0.48 | 3.95 | 1.22 | 2.80 | 3.80 | 2.54 | 0.70 | 3.03 | 3.53 | 2.07 |
| 50 | 7 | 0.59 | 9.20 | 2.53 | 4.73 | 4.45 | 3.03 | 2.21 | 5.08 | 4.24 | 2.75 |
| 51 | 7 | 0.23 | 2.92 | 1.60 | 1.69 | 1.74 | 1.74 | 0.96 | 2.17 | 2.95 | 0.79 |
| 52 | 7 | 0.93 | 2.97 | 1.17 | 1.45 | 2.58 | 1.60 | 0.48 | 1.55 | 2.22 | 0.82 |
| 53 | 7 | 0.31 | 1.51 | 0.84 | 0.86 | 1.82 | 1.95 | 0.30 | 7.32 | 0.58 | 0.58 |
| 54 | 7 | 0.33 | 0.55 | 0.43 | 0.28 | 0.23 | 0.36 | 0.23 | 0.28 | 0.09 | 0.23 |

Group 1 = Control group; Group 2 = subclinical chronic kidney disease group; Group 3 = cisplatin induced acute kidney injury group; Group 4 = cisplatin induced acute kidney injury on subclinical kidney injury group; Group 5 = ischaemia reperfusion injury on subclinical chronic kidney disease, acute group; Group 6 = ischaemia reperfusion injury on subclinical chronic kidney disease, chronic group; Group 7 = ischaemia reperfusion injury on a normal diet group. 18S = ribosomal 18S; GAPDH = Glyceraldehyde 3-phosphate dehydrogenase; ACTB = beta actin; HMBS = Hydroxymethylbilane synthase; PABPN1 = Polyadenylate-binding nuclear protein 1; HPRT = Hypoxanthine-guanine phosphoribosyltransferase; TBP = TATA binding protein; SDHA = Succinyl dehydrogenase; YWHAG = 14-3-3 protein gamma ; YWHAZ = Tyrosine 3-Monooxygenase/Tryptophan 5-Monooxygenase Activation Protein Zeta. RQ = relative gene expression quantities = $RQ_{G,k} = E_{mean, G}^{\triangle Cq_{G,k}}$, *G* = gene, *k* = sample, *∆Cq* = sample (*k*) – mean for group
