# Supplementary material for: Selection and validation of reference genes for normalisation of gene expression in ischaemic and toxicological studies in kidney disease
Source: PLoS One. 2020 May 21;15(5):e0233109. doi: 10.1371/journal.pone.0233109 (PMC7241806; doi:10.1371/journal.pone.0233109)
Supplement: S10 File — (DOCX) [file pone.0233109.s010.docx]

**Supplement 10**

**Derivation of NRQ and corresponding standard errors**

NRQ and corresponding standard error was calculated using standard statistical methods (1-4) and according to the workflow described by Hellemans and Vandesempole (5). All calculations were performed on Microsoft Excel™. Formulas for the mean amplification efficiencies and the corresponding SE_Emean_ is detailed in supplement 2. Once inter-plate calibration was completed as explained in supplement 2, Cq_E,corr_ triplicate values were averaged. The corresponding standard error (SE) was calculated according to formula 8.

Subsequently averaged Cq_E,corr,triplicate_ ($\overline{CqE,corr,triplicate}$) values were converted into efficiency corrected relative gene expression quantities (RQ) (formulas 9 to 11):

$\overline{Cq_{G}} = \frac{\sum_{1}^{54} Cq_{G,k}}{54}$ (8)

k = sample

G = gene

$\triangle Cq_{G,k}= Cq_{G,k} - \overline{Cq_{G}}$ (9)

$RQ_{G,k} = E_{mean, G}^{\triangle Cq_{G,k}}$ (10)

Normalisation factor (NF) is calculated from the geometric means of the RQs most stable reference genes.

ref = the reference genes used to calculate the NF for sample k. f = the number of reference genes used to calculate NF.

$NF_{k} = \sqrt[f]{\overset{f}{\underset{p = 1}{\prod RQ_{ref,k}}}}$ (11)

The generic formula for NRQ is stated in formula 15 and for gene of interest (GOI) KIM1 and sample k in formula 16;

$\mathrm{NRQ} = \frac{E_{GOI}^{\triangle Cq,GOI}}{\sqrt[f]{{\prod_{0}^{f} E}_{ref}^{\triangle Cq,ref}}}$ (12)

$NRQ_{KIM1,k} =\frac{RQ_{KIM1,k}}{NF_{k}}$ (13)

**References**

1. Taylor JR. An Introduction to Error Analysis: The Study of Uncertainties in Physical Measurements. Sausalito, CA: University Science Books. 1997;2nd edition.
2. David M Harrison. Error Analysis in Experimental Physical Science. 2010.
3. Farrance I, Frenkel R. Uncertainty of Measurement: A Review of the Rules for Calculating Uncertainty Components through Functional Relationships. The Clinical biochemist Reviews. 2012;33(2):49-75.
4. McCarty L. An Introduction to Measurement and Uncertainty. Sept 2006.
5. Jan Hellemans GM, Anne De Paepe, Frank Speleman and Jo Vandesompele. qBase relative quantification framework and software for management and automated analysis of real-time quantitative PCR data. Genome Biol. 2007.
