# Supplementary material for: Selection and validation of reference genes for normalisation of gene expression in ischaemic and toxicological studies in kidney disease
Source: PLoS One. 2020 May 21;15(5):e0233109. doi: 10.1371/journal.pone.0233109 (PMC7241806; doi:10.1371/journal.pone.0233109)
Supplement: S11 File — (DOCX) [file pone.0233109.s011.docx]

**Supplement 11**

***KIM-1* RT-qPCR amplification efficiency and delta Cq results of GOIs**

Mean amplification efficiency = 1.94

**S11 Table 1.**

RQ = relative gene expression quantities =

$RQ_{G,k} = E_{mean, G}^{\triangle Cq_{G,k}}$, *G* = gene, *k* = sample,

*∆Cq* = sample (*k*) – mean for group

| ***KIM-1* RQ** | ***KIM-1* RQ continued** |
| --- | --- |
| 0.09 | 0.29 |
| 0.03 | 0.09 |
| 0.05 | 0.20 |
| 0.11 | 2.01 |
| 0.10 | 11.08 |
| 0.29 | 2.78 |
| 0.65 | 7.06 |
| 0.07 | 4.44 |
| 16.15 | 1.90 |
| 20.05 | 1.28 |
| 0.03 | 6.59 |
| 0.16 | 2.20 |
| 0.15 | 5.49 |
| 0.16 | 2.09 |
| 0.30 | 1.28 |
| 0.13 | 1.87 |
| 0.56 | 1.10 |
| 0.67 | 0.07 |
| 1.00 |  |
| 0.48 |  |
| 2.49 |  |
| 0.91 |  |
| 2.71 |  |
| 1.15 |  |
| 3.35 |  |
| 8.60 |  |
| 17.68 |  |
| 10.05 |  |
| 8.64 |  |
| 12.20 |  |
| 7.39 |  |
| 0.77 |  |
| 4.15 |  |
| 0.38 |  |
| 1.86 |  |
| 4.39 |  |

***HIF1α* RT-qPCR amplification efficiency and delta Cq results**

Mean amplification efficiency = 2.00

**S11 Table 2.**

| ***HIF1α* RQ** | ***HIF1α* RQ continued** |
| --- | --- |
| 2.303651293 | -1.146431114 |
| 3.326078362 | -0.780752886 |
| 3.598816918 | -9.889811236 |
| 1.262985524 | 1.091708622 |
| 1.483238086 | 4.072141909 |
| 1.211040696 | 3.760243541 |
| -0.193047408 | 4.072141909 |
| -0.328923628 | 0.232583006 |
| 1.118243637 | -0.454651161 |
| -1.080516598 |  |
| 1.118243637 |  |
| -1.080516598 |  |
| -1.161637662 |  |
| -0.820172297 |  |
| 0.113166838 |  |
| 0.045840175 |  |
| 0.551110349 |  |
| -0.116260676 |  |
| 0.488278159 |  |
| 0.521700419 |  |
| 1.079404213 |  |
| -1.031451291 |  |
| 2.011420439 |  |
| 2.350324424 |  |
| -0.743775576 |  |
| -3.675519765 |  |
| -3.675519765 |  |
| -3.82176833 |  |
| -2.582045112 |  |
| -2.105055086 |  |
| -0.896358869 |  |
| 1.352897234 |  |
| 1.173119344 |  |
| -1.146431114 |  |
| -0.780752886 |  |
| -0.780752886 |  |
| -1.146431114 |  |
| -0.780752886 |  |

***PECAM1* RT-qPCR amplification efficiency and delta Cq results**

Mean amplification efficiency = 1.97

**S11 Table 3.**

| ***PECAM1* RQ** | ***PECAM1* RQ continued** |
| --- | --- |
| 2.019286 | -1.83305 |
| 2.76851 | -1.58652 |
| 5.252209 | 0.086441 |
| 1.252409 | 0.854926 |
| 1.412231 | 3.402369 |
| 2.999806 | 3.356773 |
| -0.02244 | -1.61752 |
| -0.46288 | -1.90855 |
| 0.71894 | -1.65314 |
| -0.78237 |  |
| -0.78237 |  |
| 0.71894 |  |
| -1.03391 |  |
| -0.7764 |  |
| -0.26918 |  |
| -0.64087 |  |
| -0.18566 |  |
| -0.23651 |  |
| -0.11389 |  |
| 0.007115 |  |
| 1.335358 |  |
| -0.8276 |  |
| 1.759771 |  |
| 1.976611 |  |
| 1.469841 |  |
| -1.14079 |  |
| -3.71527 |  |
| -3.56352 |  |
| -2.92161 |  |
| -2.09209 |  |
| -0.99641 |  |
| 0.826089 |  |
| 0.301112 |  |
| -1.82787 |  |
| 3.36894 |  |
| -1.82787 |  |
| -0.93524 |  |
| -1.47798 |  |
| -0.65614 |  |

***TGFβ1* RT-qPCR amplification efficiency and delta Cq results**

Mean amplification efficiency = 2.00

| ***TGFβ1* RQ** | ***TGFβ1* RQ continued** |
| --- | --- |
| 1.475441981 | -1.012263925 |
| 2.361431357 | 1.509176073 |
| 1.880954072 | 0.460255642 |
| 0.637533508 | 1.705931466 |
| -0.0365547 | 2.174107762 |
| 0.06554139 | 2.774077329 |
| 0.554838118 | 2.774077329 |
| 0.532249672 | 0.607336407 |
| 1.783982345 | -1.09296134 |
| -0.12739707 |  |
| -0.12739707 |  |
| 1.783982345 |  |
| -0.399124498 |  |
| 0.127382855 |  |
| 0.6142049 |  |
| 0.390639396 |  |
| 0.240903245 |  |
| 0.039708454 |  |
| 0.01241722 |  |
| 0.891308349 |  |
| 2.324917867 |  |
| 0.410941053 |  |
| 1.616760613 |  |
| -2.14121668 |  |
| 0.617987932 |  |
| -2.891108706 |  |
| -2.560849627 |  |
| -2.14121668 |  |
| -2.444133322 |  |
| -1.145103446 |  |
| -0.171909615 |  |
| 0.582585459 |  |
| 0.212554823 |  |
| -3.393533617 |  |
| -1.577690199 |  |
| -3.393533617 |  |
| -3.393533617 |  |
| -1.577690199 |  |
| -1.536011039 |  |

**S11 Table 4.**
